# Prevalence and characteristics of acute ischemic stroke and intracranial hemorrhage in patients with immune thrombocytopenic purpura and immune thrombotic thrombocytopenic purpura: a systematic review and meta-analysis

**DOI:** 10.1186/s42466-025-00374-3

**Published:** 2025-03-17

**Authors:** Syed Ameen Ahmad, Olivia Liu, Amy Feng, Andrew Kalra, Apurva Dev, Marcus Spann, Aaron M. Gusdon, Shruti Chaturvedi, Sung-Min Cho

**Affiliations:** 1 Division of Neurosciences Critical Care and Cardiac Surgery, Departments of Neurology, Surgery, Anesthesiology and Critical Care Medicine, Johns Hopkins University School of Medicine, 600 N. Wolfe Street, Phipps 455, Baltimore, MD 21287 USA; 2 Division of Cardiac Surgery, Department of Surgery, Johns Hopkins University School of Medicine, Baltimore, MD USA; 3 Sidney Kimmel Medical College, Thomas Jefferson University, Philadelphia, PA USA; 4 Informationist Services, Johns Hopkins School of Medicine, Baltimore, MD USA; 5 Division of Neurocritical Care, Department of Neurosurgery, McGovern School of Medicine, University of Texas Health Science Center, Houston, TX USA; 6 Division of Hematology, Department of Medicine, School of Medicine, Johns Hopkins University, Baltimore, MD USA

## Abstract

**Background:**

There is an emerging understanding of the increased risk of stroke in patients with immune thrombocytopenic purpura (ITP) and immune thrombotic thrombocytopenic purpura (iTTP). We aimed to determine the prevalence and characteristics of acute ischemic stroke (AIS) and intracranial hemorrhage (ICH) in patients with ITP and iTTP in a systematic review and meta-analysis.

**Methods:**

We used PubMed, Embase, Cochrane, Web of Science, and Scopus using text related to ITP, iTTP, stroke, AIS, and ICH from inception to 11/3/2023. Our primary outcome was to determine prevalence of AIS and/or ICH in a cohort of ITP or iTTP patients (age > 18). Our secondary outcomes were to determine stroke type associated with thrombopoietin receptor agonists (TPO-RAs) in ITP patients, as well as risk factors associated with stroke in ITP and iTTP patients.

**Results:**

We included 42 studies with 118,019 patients (mean age = 50 years, 45% female). Of those, 27 studies (*n* = 116,334) investigated stroke in ITP patients, and 15 studies (*n* = 1,685) investigated stroke in iTTP patients. In all ITP patients, the prevalence of AIS and ICH was 2.1% [95% Confidence Interval (CI) 0.8-4.0%] and 1.5% (95% CI 0.9%-2.1%), respectively. ITP patients who experienced stroke as an adverse event (AE) from TPO-RAs had an AIS prevalence of 1.8% (95% CI 0.6%-3.4%) and an ICH prevalence of 2.0% (95% CI 0.2%-5.3%). Prevalence of stroke did not significantly differ between all ITP patients and those treated with TPO-RAs. iTTP patients had a prevalence of AIS and ICH of 13.9% (95% CI 10.2%-18.1%) and 3.9% (95% CI 0.2%-10.4%), respectively. Subgroup analysis revealed the prevalence of AIS and ICH was greater in iTTP patients vs. all ITP patients (*p* < 0.01 and *p* = 0.02, respectively). Meta-regression analysis revealed none of the collected variables (age, sex, history of diabetes or hypertension) were risk factors for stroke in all ITP patients, although there were high levels of data missingness.

**Conclusions:**

Prevalence of different stroke types was lower in all ITP patients vs. iTTP patients. Additionally, ITP patients experienced a similar prevalence of stroke regardless of if they were specifically denoted to have been treated with TPO-RAs or not, supporting the continued use of TPO-RAs in management. Risk factors for stroke remain unclear, and future studies should continue to investigate this relationship.

**Supplementary Information:**

The online version contains supplementary material available at 10.1186/s42466-025-00374-3.

## Introduction

Immune thrombocytopenic purpura (ITP) and immune thrombotic thrombocytopenic purpura (iTTP) are hematologic disorders characterized by distinct immunologic mechanisms that lead to thrombocytopenia and an increased risk of bleeding and clotting complications [1]. In ITP, development of anti-platelet autoantibodies results in destruction of platelets and severe thrombocytopenia (platelet count < 100 × 10^9^/L) [1, 2]. In contrast, iTTP involves the formation of auto-antibodies that inhibit normal regulation of thrombus formation, leading to over-activation of platelets that results in microthrombi that obstruct blood flow in small vessels [1]. Both conditions can lead to severe complications such as stroke.

Thrombocytopenia is a known risk factor for intracranial hemorrhage (ICH) [3]. While the association between ITP and ICH has been well documented in pediatric populations [4–6], data on this relationship in adults remain sparse [7, 8]. Additionally, the frequency of acute ischemic stroke (AIS) is less well characterized. One meta-analysis (*n* = 12,442 patients) demonstrated an increased risk of thromboembolism in patients with ITP, but did not specifically identify the number of AIS occurrences [9]. While another systematic review (*n* = 88,442 patients) revealed that patients with ITP had an increased risk of AIS and transient ischemic attacks (TIAs), they did not investigate the prevalence of ICH [10]. Moreover, insight into ITP treatments and their association with stroke is limited. While initial management involves steroids, patients with suboptimal responses receive treatments such as receptor agonists (TPO-RAs), rituximab, or splenectomy [11]. While the TPO-RAs are effective in raising platelet counts, they have also been shown to be linked with adverse events (AEs), such as stroke. While meta-analyses on this topic report bleeding complications or cardiovascular events broadly, specific insights into stroke subtype and prevalence remain limited [13–16].

Data on stroke in iTTP are even more limited. A case of iTTP associated ICH (confirmed by brain magnetic resonance imaging (MRI)) has previously been reported [17]. In regard to AIS, an analysis of 26 case reports demonstrated that AIS was the initial manifestation of iTTP [18]. Recent data suggests that iTTP survivors have nearly five-fold increased risk of stroke compared to age and sex matched controls [19]. However, to our knowledge, there has not been a systematic review on the topic of stroke development in iTTP.

Overall, despite being well recognized clinically, stroke in patients with ITP and iTTP remains poorly characterized. Limited data exist on the prevalence of different stroke types, risk factors, and interventions that are associated with strokes in ITP and iTTP. Therefore, we aimed to systematically review published studies that report on stroke occurrence in patients with ITP and iTTP to [1] determine the prevalence of AIS and/or ICH; [2] assess stroke as an AE due to TPO-RAs in patients with ITP; and [3] analyze the risk-factors associated with stroke.

## Methods

This systematic review was reported in accordance with the 2020 Preferred Reporting Items for Systematic Reviews and Meta-Analyses (PRISMA) guidelines [20]. Institutional review board approval was not required because this study represents a secondary analysis of aggregated public datasets and did not directly involve human subjects.

### Search strategy

Eligible studies were identified from systematic searches in MEDLINE (PubMed), Embase, Cochrane, Web of Science, and Scopus without language restriction from inception to 11/3/2023. Controlled vocabulary, such as Medical Subject Headings and Emtree terms, when appropriate, were used in combination with keywords for the concepts of ITP, iTTP, stroke, AIS, and ICH. The studies were then independently reviewed and evaluated by our team (O.L. and A.F.) for eligibility using Covidence. Conflicts were resolved by a third reviewer (S.A.A.). Articles that met the inclusion criteria were obtained and reviewed. References of the included articles were also screened for additional studies (i.e., “citation searching”). The full search strategy can be found in the Appendix 1.

### Inclusion and exclusion criteria

Studies were eligible if they (1) reported frequency of AIS and/or ICH events in either ITP or iTTP patients and (2) were in a cohort design, a cross-sectional design, a randomized controlled trial, or a case series including five or more cases. Studies were excluded if they did not contain information regarding the number of AIS and/or ICH events in ITP or iTTP patients, or if prevalence could not be calculated. Cohorts focusing on secondary ITP were excluded. Studies that did not separate AIS from TIA occurrence were excluded since the primary focus was on AIS. Studies that reported the number of hospitalizations/discharges (rather than the number of patients) in which a stroke occurred were excluded due to the possibility of a single patient experiencing multiple hospitalizations. These articles were discussed among authors before being excluded. Abstracts without corresponding full texts such as conference proceedings, case series < 5 patients, editorials, commentaries, systematic reviews and meta-analyses, narrative reviews, studies with pediatric/neonatal patients (younger than 17 years) were excluded.

### Study selection and data extraction

Each study’s title and abstract were independently screened by two reviewers (O.L. and A.F.) in Covidence. Disagreements were resolved by a third reviewer (S.A.A.). Full texts of potential studies were then reviewed by three reviewers (O.L., A.F., and S.A.A.). Data were extracted into an Excel spreadsheet (Microsoft Corp., Redmond, WA). Study characteristics that were extracted included study design, sample size, patients’ demographics and characteristics [age, sex, body mass index (BMI), and comorbidities—including cardiovascular disease, smoking history, chronic kidney disease (CKD), diabetes, hypertension (HTN), and hyperlipidemia (HLD), as defined by each study], platelet count, management of ITP/iTTP [including steroids, intravenous immune globulin (IVIG), non-steroidal Anti-Inflammatory Drugs (NSAIDs), splenectomy, anticoagulants, and platelet transfusions], number of AIS and/or ICH events in the cohort, and type of TPO-RA if the study was looking at drug AEs for patients with ITP. Extractions were independently conducted and discussed if discrepancies arose.

### Definitions and outcomes

Our primary outcome was to determine the prevalence of AIS and/or ICH in a cohort of either ITP or iTTP patients. Our secondary outcome was to explore the prevalence/type of stroke reported as an AE in ITP patients treated with TPO-RAs. Additional secondary outcomes included assessing the risk factors (sex, age, presence of comorbidities) associated with stroke development. Our ITP group was not delineated based on TPO-RA administration, however, subgroup analysis was conducted on ITP patients who were specifically denoted to have been treated with TPO-RAs. Stroke development in this subgroup was labeled as an AE in the aftermath following drug administration. TPO-RAs included use of eltrombopag, romiplostim, and rhTPO. All iTTP patients in this study experienced stroke independent from the use of any drug treatments. Our definition of AIS followed the definition of AIS used in each study and did not include TIAs or hypoxic-ischemic brain injury. Meaning—if a study did not clearly differentiate between AIS and TIA or the reported cerebral events were ambiguous (not clearly an ischemic stroke), it was excluded from our study. ICH was defined as including intracerebral hemorrhage, subarachnoid hemorrhage, epidural hemorrhage, intraparenchymal hemorrhage, and subdural hemorrhage. Total stroke was defined as combining both AIS and ICH.

### Risk of bias assessment

Two investigators (A.F. and A.D.) independently reviewed and evaluated the risk of bias of each included study. The Newcastle-Ottawa scale was used to assess the quality of case-control and cohort studies [21]. Patient selection, comparability, and assessment of outcome or exposure were the three domains of the Newcastle-Ottawa scale. The Cochrane risk-of-bias tool 2 (RoB 2) was used to assess the quality of randomized controlled trials [23]. Scores ranging from 0 to 9 points were assigned to each included study. Lower points indicate high risk of bias, while high-quality studies had 6 points or more. Any disagreements were resolved by a third investigator (O.L.).

### Statistical analysis

The statistical analysis was developed via previously reported methods [23]. Categorical variables were reported as raw numbers and percentages. Weighted mean and standard deviation (SD) calculations were performed for continuous variables related to patient’s demographics and clinical characteristics. The prevalence of AIS and ICH was reported in each study based on the number of patients with the outcome divided by the number of ITP or iTTP patients (raw number calculation). Subgroup analysis comparing the development of AIS, ICH, and total stroke in ITP patients vs. iTTP patients was carried out. Subgroup analysis comparing the development of either AIS or ICH in ITP patients not treated with TPO-RAs vs. ITP patients treated with TPO-RAs was also conducted.

Random-effects models with the inverse variance method were used for meta-analyses of the prevalence of each outcome due to substantial heterogeneity between studies because random-effects meta-analyses permit for heterogeneity through the assumption that underlying effects correspond to a normal distribution; the Freeman-Tukey double-arcsine transformation was used for all meta-analyses. For the between-study variance tau^2^, the Sidik-Jonkman estimator was used [24, 25], and the Hartung-Knapp method was used for adjustment of confidence intervals [26]. The Cochrane *Q* statistic (χ^2^ test) was used to test for the presence of heterogeneity, and the magnitude of the heterogeneity was assessed with the *I*^2^ statistic, ranging from 0 to 100% [27].

Meta-regression with pre-specified variables (including age, sex, history of diabetes and HTN) was performed with the prevalence of AIS, ICH, or total stroke as the response variable in ITP and iTTP populations separately. All statistical analyses were performed using R Studio (R *version*, www.r-project.org*).*

### Standard protocol approvals, registrations, and patient consents

The study protocol was registered on PROSPERO (CRD42023468463). No institutional review board approval was required for this study because it uses deidentified data.

### Data availability

All data used in this manuscript are deidentified and publicly available.

## Results

**Fig. 1:**
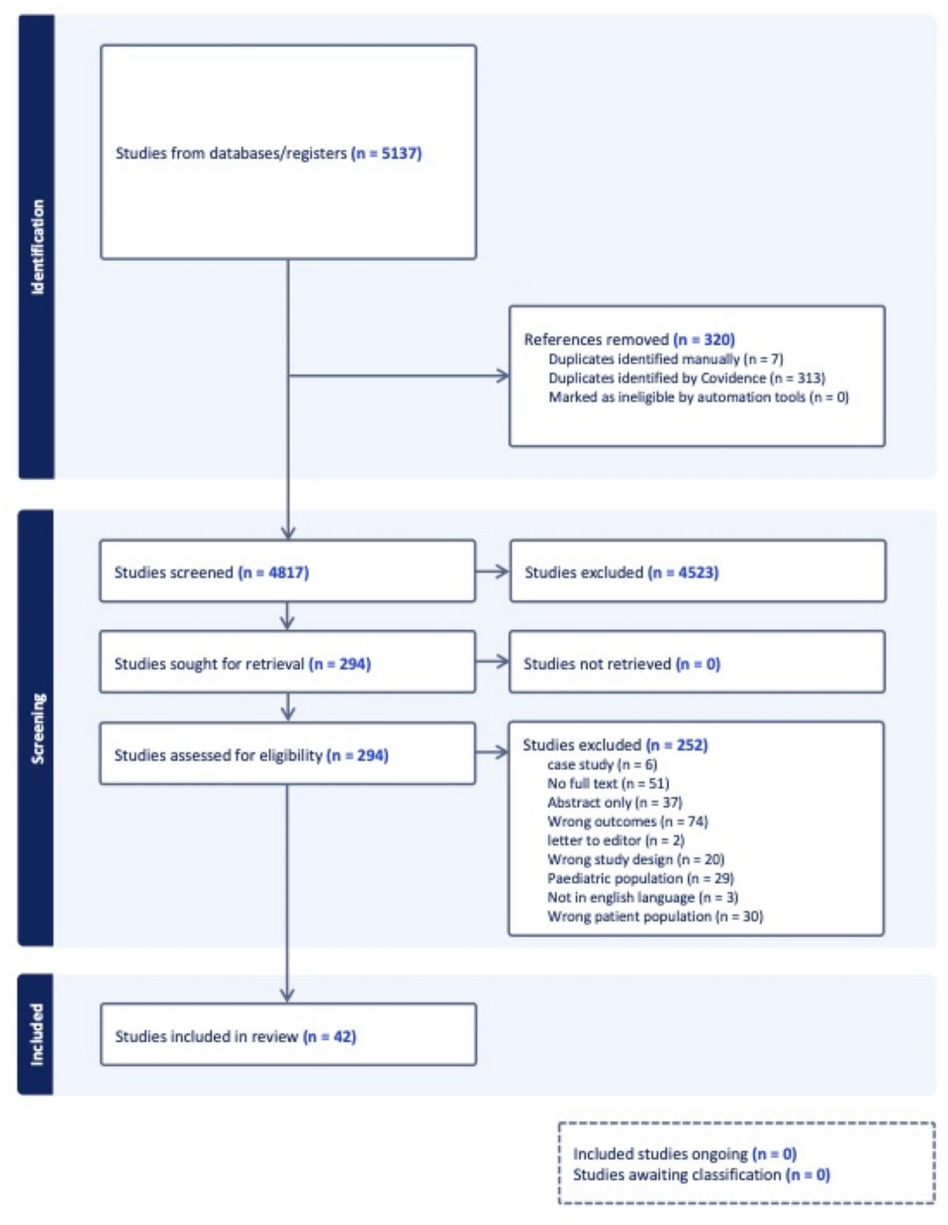
PRISMA flowchart for the creation of our study cohort

Our search identified 4,817 studies, yielding 294 articles that were assessed for full-text availability and eligibility. Of these, 42 studies (*n* = 118,109 patients) were included in our review (Fig. 1). 27 studies (*n* = 116,334) reported stroke in ITP patients (including studies specifically looking at TPO-RA administration), 8 studies (*n* = 4,390) investigated stroke as an AE in ITP patients treated with TPO-RAs, and 15 studies (*n* = 1,685) investigated stroke in iTTP patients. In all 42 studies, the weighted average age was 50.33 (Standard Deviation (SD) = 1.66) years and the weighted average BMI was 25.20 (SD = 0.40) kg/m^2^. There were 53,335 females (45%) and 38.930 males (33%), with sex not reported for 25,754 (22%) patients.

### Bias assessment

The overall median risk of bias was approximately 8 (Interquartile Range: 7–9), indicating little risk of bias across all included observational studies (Appendix 2). Among the 4 randomized controlled trials, the RoB 2 tool showed high risk of bias for 3 studies and low risk of bias for 1 study (Appendix 3).

### ITP cohort characteristics

Of the total 116,334 patients with ITP, the weighted average age was 50.44 (SD = 2.04) years old and the weighted average BMI was 24.3 (SD = 1.90) kg/m^2^. Average platelet count was 67.60 × 10^9^ (SD = 4.26 × 10^9^ U/L). The most reported comorbidity was HTN (17,880; 16%), followed by HLD (10,353; 9%), diabetes mellitus (8,729; 8%), and any history of smoking (5,758, 5%). Included studies reported receipt of splenectomy (925; 0.8%), anticoagulants (65; 0.05%), steroids (571; 0.5%), and platelet transfusions (105; 0.09%)—although there were high levels of data missingness (Table 1). Amongst the 4,390 ITP patients treated with TPO-RAs, the weighted average age was 41.86 (SD = 1.53) years, and the weighted average BMI was 29.1 (SD = 7.0 kg/m^2^). Other demographic information for this cohort can be found in Supplementary Table 1.

**Table 1:** Clinical and demographic characteristics of patients with ITP and iTTP

Variable	ITP (*n* = 116,334)	iTTP (*n* = 1,685)	Total (*n* = 118,019)
Age (years)	50.44 (2.04)	47.52 (4.51)	50.33 (1.66)
BMI (kg/m^2)	24.3 (1.90)	28.27 (0.14)	25.20 (0.40)
Sex			
Female	52,349 (45%)	986 (59%)	53,335 (45%)
Male	38,414 (33%)	516 (31%)	38,930 (33%)
Not reported	25,571 (22%)	183 (10%)	25,754 (22%)
Platelet Count (10^9 U/L)	67.60 (4.26)	47.54 (10.37)	66.76 (10.42)
Medical History			
Cardiovascular Disease	2,510 (2%)	3 (0.2%)	2,513 (2%)
Smoking	5,758 (5%)	24 (1%)	5,782 (5%)
Chronic Kidney Disease	1,726 (1%)	79 (5%)	1,805 (1%)
COPD	2,300 (2%)	0 (0%)	2,300 (2%)
Diabetes	8,729 (8%)	94 (6%)	8,823 (8%)
Hypertension	17,880 (15%)	218 (12%)	18,098 (15%)
Hyperlipidemia	10,353 (9%)	78 (5%)	10,431 (9%)
Dyslipidemia	47 (0.05%)	24 (1%)	71 (0.06%)
Congestive Heart Failure	3,908 (3%)	0 (0%)	3,908 (3%)
Atrial Fibrillation	3,626 (3%)	27 (2%)	3,653 (3%)
Treatment received for ITP/iTTP			
Splenectomy	925 (0.8%)	0 (0%)	925 (0.8%)
Anti-coagulants	65 (0.05%)	0 (0%)	65 (0.05%)
Steroids	571 (0.5%)	206 (12%)	777 (0.7%)
Platelet Transfusion	105 (0.09%)	15 (1%)	120 (0.1%)
Plasmapheresis	0 (0%)	217 (13%)	217 (0.2%)
Caplacizumab	0 (0%)	35 (2%)	35 (0.02%)

BMI: Body mass index, COPD: Chronic Obstructive Pulmonary Disease, ITP: Immune thrombocytopenic purpura, iTTP: Thrombotic thrombocytopenic purpura. Continuous variables reported as mean (standard deviation). Categorical variables count (percentage)

### Stroke prevalence in all ITP patients

**Fig. 2:**
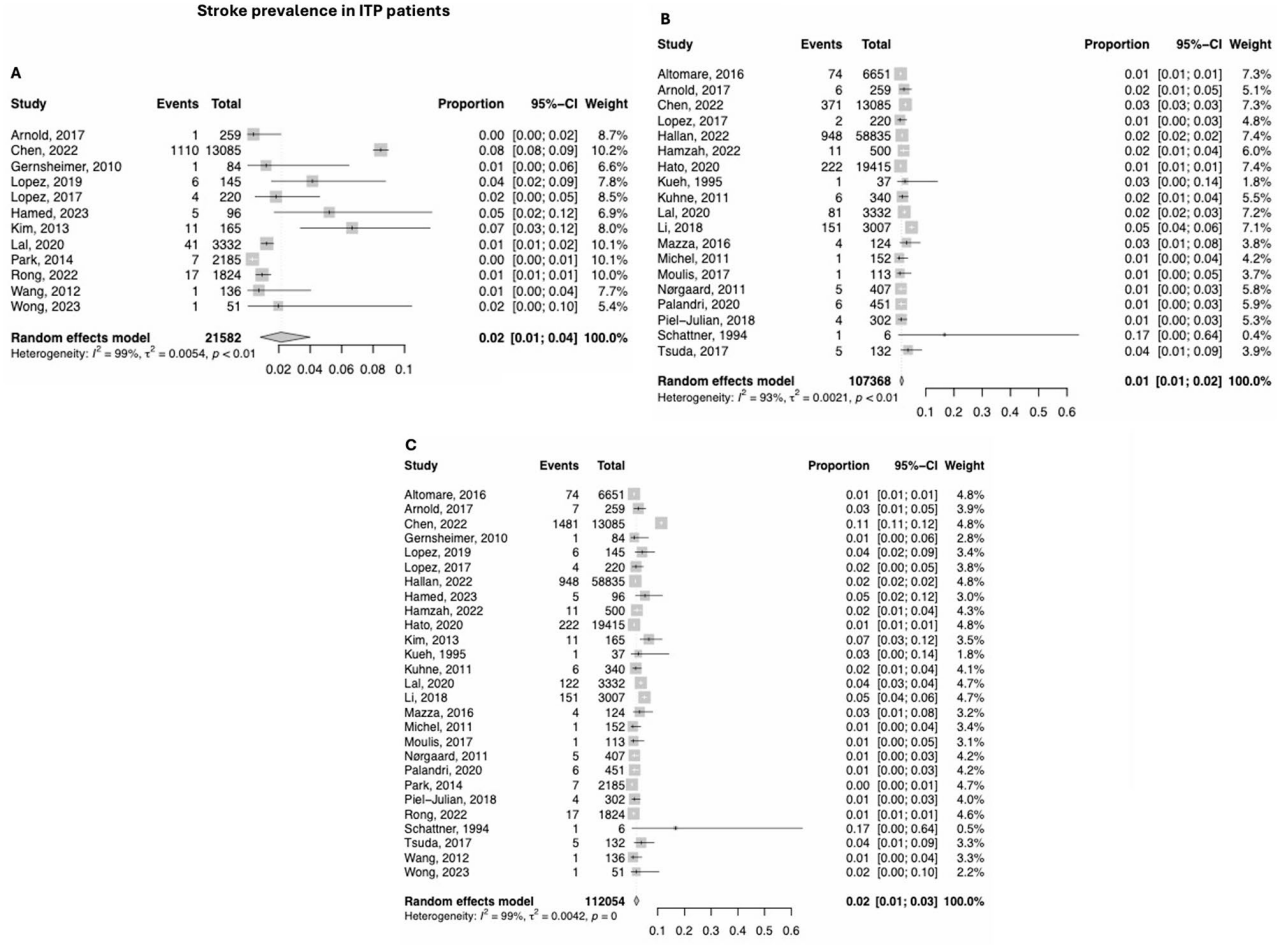
Forest plots of the prevalence of stroke in studies on ITP patients **A**). Prevalence of AIS in ITP patients. **B**). Prevalence of ICH in ITP patients. **C**). Prevalence of both AIS and ICH in ITP patients. CI = Confidence Interval

In all ITP patients, the prevalence (*n* = 116,334) of AIS was 2.1% [95% Confidence Interval (CI) 0.8-4.0%] and the prevalence of ICH was 1.5% (95% CI 0.9%-2.1%) (Fig. 2A and B). The prevalence of total stroke was 2.0% (95% CI 1.2%-2.9%) (Fig. 2C).

### TPO-RA associated stroke in ITP

**Fig. 3:**
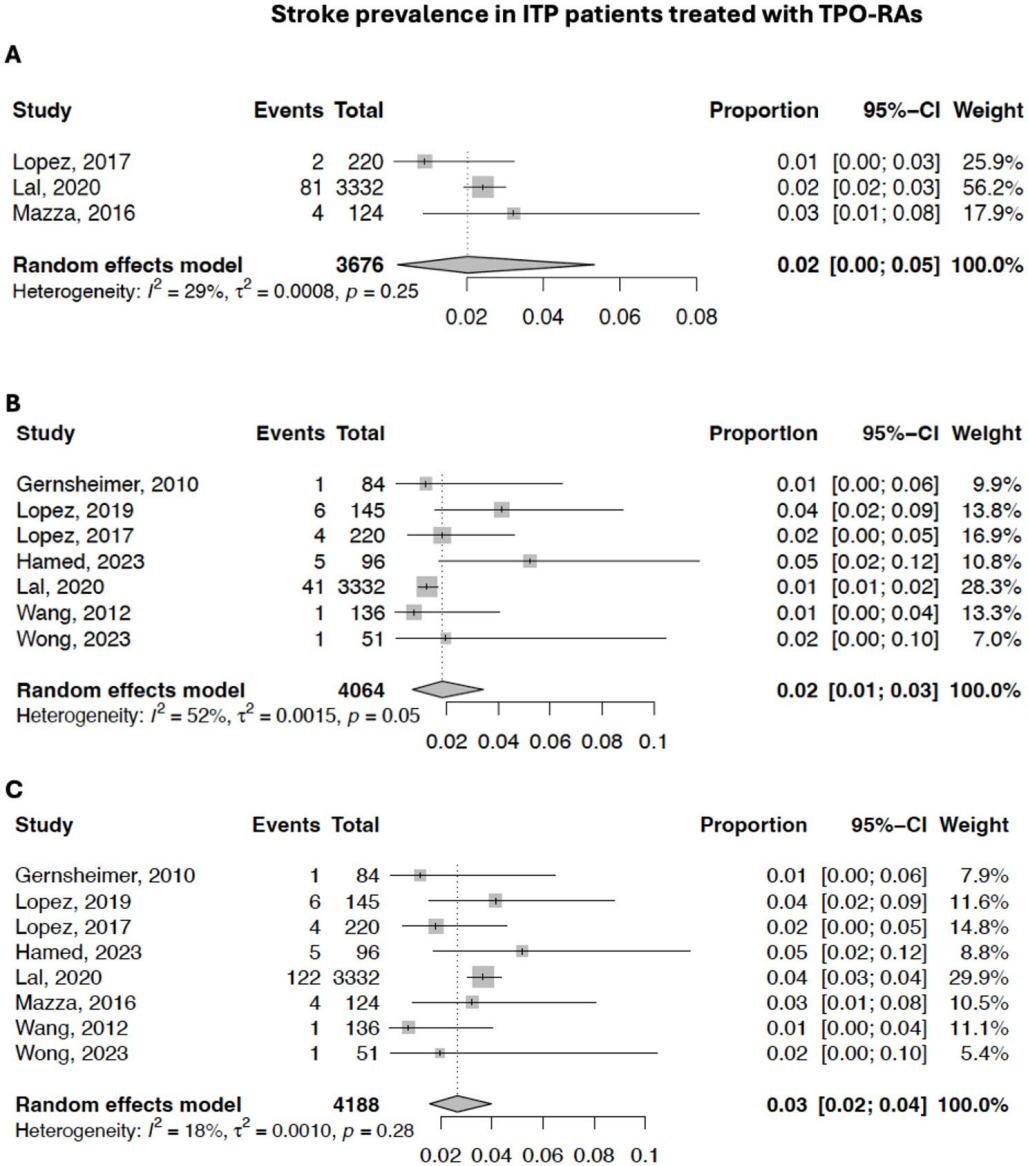
Forest plots of the prevalence of stroke as an adverse event in studies on ITP patients treated with TPO-RAs. **A**). Prevalence of ICH **B**). Prevalence of AIS. **C**). Prevalence of both AIS and ICH. CI = Confidence Interval

There were 2 studies (*n* = 135) that investigated romiplostim alone, 2 (*n* = 365) that investigated eltrombopag alone, 3 (*n* = 3,754) that investigated both romiplostim and eltrombopag, and 1 (*n* = 136) that investigated rhTPO. In our analysis of 4,390 patients, 9 patients on eltrombopag experienced AIS and 17 patients experienced ICH. A total of 5 patients on romiplostim experienced AIS and 2 experienced ICH. The overall prevalence of AIS, ICH, and total stroke was 1.8% (95% CI = 0.6-3.4%), 2.0% (95% CI = 0.2-5.3%), and 2.6% (95% CI = 1.5-4.0%) (Fig. 3).

### Comparison of stroke prevalence in ITP patients based on TPO-RA administration

The prevalence of AIS, ICH, and total stroke was not significantly different in ITP patients treated with TPO-RAs when compared to ITP patients not treated with TPO-RAs (*p* = 0.88, *p* = 0.79, and *p* = 0.37, respectively).

### iTTP cohort characteristics

Of the 1,685 stroke patients with iTTP analyzed, the weighted average age was 47.52 ​​(SD = 4.51) years, and the weighted average BMI was 28.20 (SD = 0.14 kg/m^2^). Average platelet count was 47.54 × 10^9^ (SD = 10.37 U/L). The most reported comorbidity was HTN (218; 12%), followed by diabetes (94; 6%), and chronic kidney disease (79; 5%). Studies reported receipt of steroids (206; 12%), platelet transfusions (15, 1%), plasmapheresis (217; 13%), and capacizumab (35; 2%). (Table 1).

### Stroke prevalence in iTTP patients

**Fig. 4:**
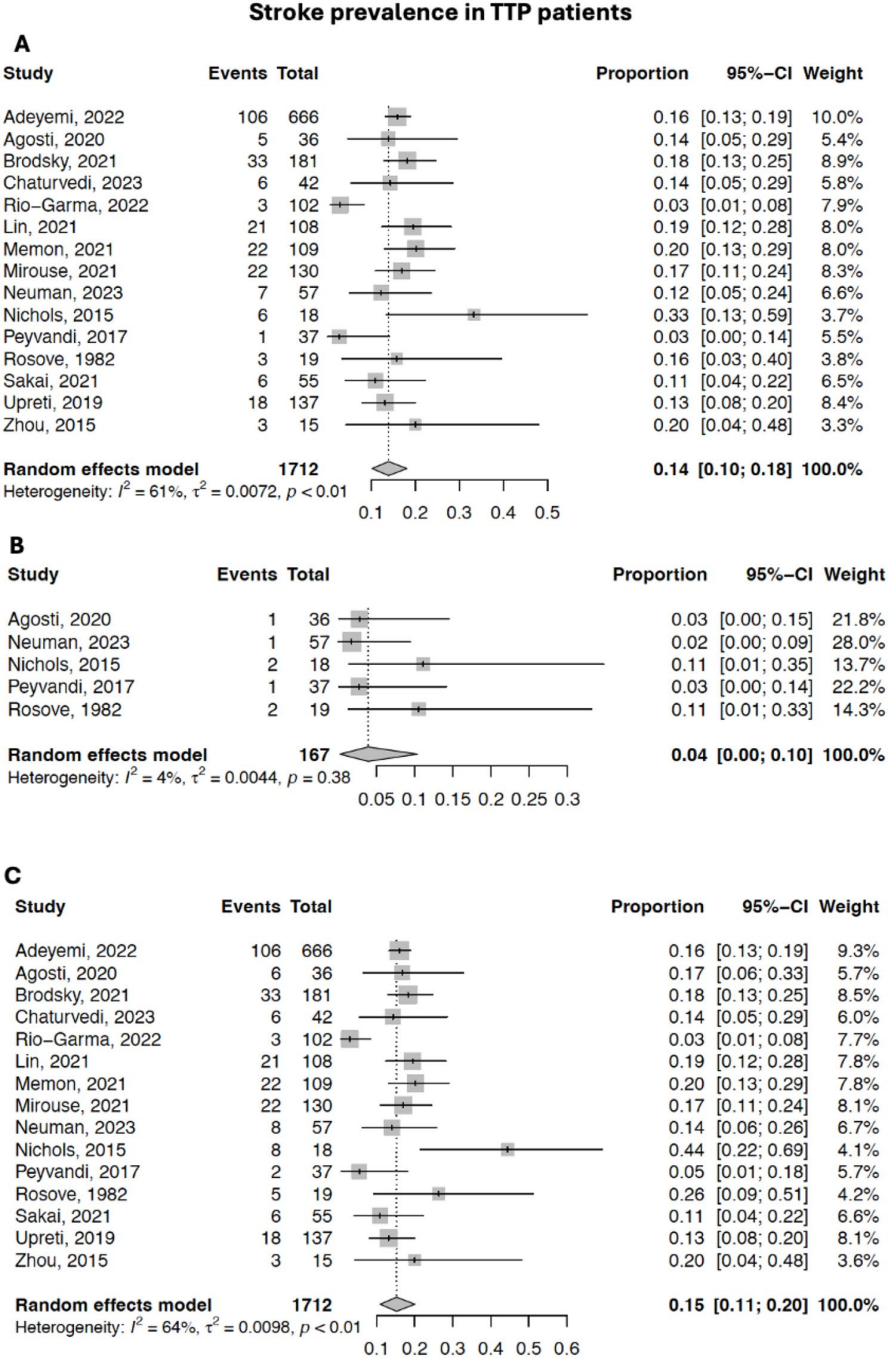
Forest plots of the prevalence of stroke in studies on iTTP patients. **A**). Prevalence of AIS in iTTP patients. **B**). Prevalence of ICH in iTTP patients. **C**). Prevalence of both AIS and ICH in iTTP patients. CI = Confidence Interval

In iTTP patients, the prevalence (total patient *n* = 1,685) of AIS was 13.9% (95% CI 10.2%-18.1%) and the prevalence of ICH was 3.9% (95% CI 0.2%-10.4%) (Fig. 4A and B). The prevalence of total stroke among iTTP patients was 15.2% (95% CI 10.9%-20.1%) (Fig. 4C).

### Exploratory analysis: strokes in ITP vs. iTTP

When comparing stroke prevalence in all ITP patients vs. iTTP patients in subgroup analysis, the prevalence of AIS, ICH, and total stroke was significantly higher in iTTP patients compared to ITP patients (*p* < 0.01, *p* = 0.02, and *p* < 0.01).

### Risk factors

Meta-regression analysis revealed that none of the tested variables (sex, age, presence of HTN and diabetes mellitus) were significant risk factors for the development of total stroke in ITP patients (Supplementary Table 2). Other variables (such as platelet count, use of steroids, IVIG, NSAIDs, or anticoagulants, and presence of splenectomy or platelet transfusions) were not included in the model due to data missingness. Data missingness, as well as lack of power, also limited our ability to conduct meta-regression analysis in iTTP patients.

## Discussion

Herein, we performed a comprehensive systematic review and meta-analysis that evaluated the prevalence of stroke in adult patients with ITP and iTTP. AIS (2.1%) was a more frequent complication than ICH (1.5%) in ITP patients, whereas iTTP patients had a significantly higher prevalence of AIS (13.9%) and ICH (3.9%) compared to ITP patients. Notably, stroke prevalence did not differ significantly between ITP patients treated with TPO-RAs and the overall ITP cohort, suggesting no increased stroke risk associated with TPO-RA use.

Our results (AIS = 2.1%) support the notion that ITP associated AIS is not an uncommon complication. The mechanism for thrombosis leading to AIS may best be explained by the presence of platelet microparticles (PMPs). The development of AIS in ITP patients may be explained by platelet microparticles (PMPs), which contribute to atherosclerosis, plaque rupture, and ischemia [30, 31]. Studies have shown that ITP patients with AIS have significantly higher PMP levels than healthy controls [30]. In addition, ITP is associated with a prothrombotic phenotype due to elevated coagulation factors and preactivated platelets [13]. Certain treatments, such as splenectomy and long-term corticosteroid use, may further amplify this risk. Finally, patients with ITP may have coexisting autoimmune disease such as systemic lupus erythematosus or antiphospholipid syndrome that also increase the risk of thrombotic events such as stroke.

AIS (1.8%) and ICH (2.0%) prevalence in ITP patients treated with TPO-RAs was comparable to the overall ITP cohort, suggesting no significant increase in stroke risk due to TPO-RAs. These drugs work by activating TPO receptors on megakaryocytes, hematopoietic stem cells, and platelets to stimulate platelet production [12]. Although there has been concern regarding thrombosis associated with TPO-RAs, there has been no clear evidence to suggest an increased risk [32]. Our data supports this notion, and TPO-RAs should continue to be used as a mainstay in ITP treatment from a thromboembolic perspective. However, in clinical practice, extra consideration for comorbidities should still be given when considering high-risk patients.

The prevalence of ICH in all ITP patients (1.5%) in our study is in accordance with what was reported in a prior systematic review on a similar topic [*n* = 1,896 patients, weighted proportion of 1.4% [28]. ITP may predispose patients to ICH due to the fact that a low platelet count can lead to disruption of vascular hemostasis [29], resulting in excess bleeding. In accordance with this, our cohort of ITP patients had a greater average platelet count than iTTP patients and experienced a significantly lower prevalence of ICH. However, the data is not completely conclusive, as a prior systematic review reported no consistent association between bleeding and platelet count [28]. For this reason, more investigation is needed to determine the link, if any, between platelet count and ICH risk.

Taken together, patients with ITP are at a paradoxical increased risk for both AIS and ICH. Currently, there is no formal consensus on how these subtypes of strokes should be managed, however, both factors should be weighed and considered when treating patients with ITP. While management strategies for stroke focus on vital sign and cardiac monitoring, routine labs, and neurological examinations, these may hold less utility for ITP and iTTP patients with a hematological basis for their strokes. For this reason, our data give evidence that monitoring strategies should be developed with respect to stroke in ITP and iTTP.

Focusing on iTTP, the prevalence of AIS was significantly greater in this patient population when compared to all ITP patients (13.9% vs. 2.1%, *p* < 0.02). This association may be explained by the formation of diffuse microthrombi resulting in AIS [33]. The role of antithrombotic therapy in iTTP remains controversial. While these agents may reduce thrombotic complications [34–36], they also pose a bleeding risk, particularly given the 3.9% ICH prevalence observed in our study. Despite the high AIS prevalence (13.9%), only 2.3% of iTTP patients in our dataset received antiplatelet therapy, and none received anticoagulants. This may reflect physician hesitancy due to bleeding concerns. However, emerging evidence suggests that carefully selected patients may benefit from controlled antithrombotic use. Similarly, few patients received caplacizumab (2%) or plasmapheresis (13%)—agents that can help to reduce platelet adhesion and remove autoantibodies. Further research is needed to determine whether individualized antithrombotic and antiplatelet strategies—accounting for stroke risk factors, platelet counts, and endothelial function—could safely reduce AIS risk without increasing ICH incidence.

There are several limitations to discuss in this study. First, pertinent information, such as comorbidities, management received, and outcomes (such as mortality) were not uniformly controlled across studies. This can lead to a potential residual confounding effect and limit the interpretation of our findings. This amount of data missingness limited our ability to conduct the meta-analysis regression for the risk factors of stroke. Future studies should attempt to uncover these risk factors, as they hold importance for managing patients with ITP and iTTP in the acute setting. While comparisons were made between ITP patients not treated with TPO-RAs and ITP patients treated with TPO-RAs, as well as between all ITP patients and iTTP patients, direct comparison is limited given these studies were not aimed to compare these groups. Furthermore, while we attempted to collect data on the etiology of AIS or the subtype of ICH, these data were reported sparsely. Prospective studies aimed at determining the prevalence of stroke subtypes in ITP and iTTP will help to elucidate the relevance of hematological conditions in this population of stroke patients. Another limitation was that although we took steps to only include AIS studies within the parameters of our criteria, there may have still been inherent heterogeneity in how AIS was defined across different studies. The inherent structure of the meta-analysis limits our ability to control for this variability, making it difficult to draw relevant conclusions for clinical practice. While we were able to demonstrate that stroke is a common occurrence in this population, future studies should now attempt to prospectively control for definitions like AIS in order to make results more clinically relevant. Additionally, while many of the studies analyzed stroke in the post-hospitalization period, they did not provide information on how long after stroke occurred from diagnosis/hospital onset, leading to an inability to calculate period prevalence. Finally, stroke prevalence was not able to be stratified based on the phase of ITP or iTTP. Both timing and phase of disease are clinically important variables since they can provide stratification for risk of stroke occurrence. Understanding this relationship may be paramount for management of stroke in ITP and iTTP moving forward.

## Conclusions

ITP patients experienced a similar prevalence of AIS and ICH regardless of if they were specifically denoted to have been treated with TPO-RAs or not, supporting the use of TPO-RAs in ITP management. iTTP patients experienced a significantly higher prevalence of AIS and ICH compared to all ITP patients. The high prevalence of AIS warrants further investigation about the risks and benefits associated with antiplatelets and anticoagulants as stroke prophylaxis. Future studies should investigate the timing of stroke occurrence following an ITP or iTTP diagnosis and identify the appropriate strategies for long-term management.

## Electronic supplementary material

Below is the link to the electronic supplementary material.

Supplementary Material 1

Supplementary Material 2

Supplementary Material 3

Supplementary Material 4

## Data Availability

All data generated or analyzed during this study are publically available as published articles.
