# Supplementary material for: Prevalence and characteristics of acute ischemic stroke and intracranial hemorrhage in patients with immune thrombocytopenic purpura and immune thrombotic thrombocytopenic purpura: a systematic review and meta-analysis
Source: Neurol Res Pract. 2025 Mar 17;7(1):19. doi: 10.1186/s42466-025-00374-3 (PMC11921978; doi:10.1186/s42466-025-00374-3)
Supplement: Supplementary file 2 — Supplementary Material 2 [file 42466_2025_374_MOESM2_ESM.docx]

**Appendix**

**Appendix 1: Search Terms and Search Strategy**

**Appendix 2: NOS Bias Assessment for included studies.**

**Appendix 3: The revised Cochrnae risk-of-bias for randomized tools (RoB 2)**

**Appendix 1: Search Strategy**

Search Summary

| **Topic** | Adult patients with a diagnosis of ITP or TTP and who subsequently developed either an ischemic or hemorrhagic stroke. |
| --- | --- |
| **Reference Manager** | EndNote |
| **Timeline** |  |
| **Restrictions or Limitations** | None |
| **Key Articles** |  |
| **Databases** | PubMed, Embase, Cochrane, Web of Science and Scopus |
| **Date Run** | 11/3/2023 |
| **Total Number of Results** | 8,256 |
| **Number of Duplicates Removed** | 3,119 |
| **Remaining Number of Results** | 5,137 |
| **Search Prepared By** | Marcus Spann |

# PubMed

| Concept | Query | Total |
| --- | --- | --- |
| ITP/TTP | "purpura, thrombocytopenic, idiopathic"[MeSH Terms] OR "Immune thrombocytopenic purpura"[Title/Abstract] OR "Immune Thrombocytopenia"[Title/Abstract] OR "Immune Thrombocytopenias"[Title/Abstract] OR "Werlhof Disease"[Title/Abstract] OR "Autoimmune Thrombocytopenia"[Title/Abstract] OR "Autoimmune Thrombocytopenias"[Title/Abstract] OR "Idiopathic Thrombocytopenic Purpura"[Title/Abstract] OR "Idiopathic Thrombocytopenic Purpuras"[Title/Abstract] OR "purpura, thrombotic thrombocytopenic"[MeSH Terms] OR "thrombotic thrombocytopenic purpura*"[Title/Abstract] OR "Moschcowitz Disease"[Title/Abstract] OR "Moschkowitz Disease"[Title/Abstract] OR "Upshaw Schulman Syndrome"[Title/Abstract] OR "ADAMTS13 Protein"[MeSH Terms] OR "ADAMTS13 Protein"[Title/Abstract] OR "ADAMTS13"[Title/Abstract] OR "ADAMTS 13"[Title/Abstract] OR "von Willebrand Factor Cleaving Protease"[Title/Abstract] OR "ADAMTS13 Protease"[Title/Abstract] OR "vWF Cleaving Protease"[Title/Abstract] | 21,683 |
| Stroke | "Stroke"[MeSH Terms] OR "Stroke"[Title/Abstract] OR "Strokes"[Title/Abstract] OR "cerebrovascular accident*"[Title/Abstract] OR "CVA"[Title/Abstract] OR "CVAs"[Title/Abstract] OR "Cerebrovascular Apoplexy"[Title/Abstract] OR "brain vascular accident*"[Title/Abstract] OR "Ischemic Infarct"[Title/Abstract] OR ("Intracranial Hemorrhages"[MeSH Terms] OR "Intracranial Hemorrhages"[Title/Abstract] OR "Intracranial Hemorrhage"[Title/Abstract] OR "brain hemorrhage*"[Title/Abstract]) OR ("hemorrhagic transformation*"[Title/Abstract] OR "intraparenchymal hemorrhage*"[Title/Abstract] OR "microvascular injur*"[Title/Abstract] OR "cerebral micro bleed*"[Title/Abstract] OR "neurologic deficit*"[Title/Abstract] | 451,125 |
|  |  |  |
| Sum | | |
| **"purpura, thrombocytopenic, idiopathic"[MeSH Terms] OR "Immune thrombocytopenic purpura"[Title/Abstract] OR "Immune Thrombocytopenia"[Title/Abstract] OR "Immune Thrombocytopenias"[Title/Abstract] OR "Werlhof Disease"[Title/Abstract] OR "Autoimmune Thrombocytopenia"[Title/Abstract] OR "Autoimmune Thrombocytopenias"[Title/Abstract] OR "Idiopathic Thrombocytopenic Purpura"[Title/Abstract] OR "Idiopathic Thrombocytopenic Purpuras"[Title/Abstract] OR "purpura, thrombotic thrombocytopenic"[MeSH Terms] OR "thrombotic thrombocytopenic purpura*"[Title/Abstract] OR "Moschcowitz Disease"[Title/Abstract] OR "Moschkowitz Disease"[Title/Abstract] OR "Upshaw Schulman Syndrome"[Title/Abstract] OR "ADAMTS13 Protein"[MeSH Terms] OR "ADAMTS13 Protein"[Title/Abstract] OR "ADAMTS13"[Title/Abstract] OR "ADAMTS 13"[Title/Abstract] OR "von Willebrand Factor Cleaving Protease"[Title/Abstract] OR "ADAMTS13 Protease"[Title/Abstract] OR "vWF Cleaving Protease"[Title/Abstract]) AND ("Stroke"[MeSH Terms] OR "Stroke"[Title/Abstract] OR "Strokes"[Title/Abstract] OR "cerebrovascular accident*"[Title/Abstract] OR "CVA"[Title/Abstract] OR "CVAs"[Title/Abstract] OR "Cerebrovascular Apoplexy"[Title/Abstract] OR "brain vascular accident*"[Title/Abstract] OR "Ischemic Infarct"[Title/Abstract] OR ("Intracranial Hemorrhages"[MeSH Terms] OR "Intracranial Hemorrhages"[Title/Abstract] OR "Intracranial Hemorrhage"[Title/Abstract] OR "brain hemorrhage*"[Title/Abstract]) OR ("hemorrhagic transformation*"[Title/Abstract] OR "intraparenchymal hemorrhage*"[Title/Abstract] OR "microvascular injur*"[Title/Abstract] OR "cerebral micro bleed*"[Title/Abstract] OR "neurologic deficit*"[Title/Abstract]))** | | **834** |
| Number of Citations after Deduplication |  | **834** |

# Embase

| Concept | Query | Total |
| --- | --- | --- |
| ITP/TTP | 'idiopathic thrombocytopenic purpura'/exp OR 'immune thrombocytopenic purpura':ab,ti OR 'immune thrombocytopenia':ab,ti OR 'immune thrombocytopenias':ab,ti OR 'werlhof disease':ab,ti OR 'autoimmune thrombocytopenia':ab,ti OR 'autoimmune thrombocytopenia'/exp OR 'idiopathic thrombocytopenic purpura':ab,ti OR 'idiopathic thrombocytopenic purpuras':ab,ti OR 'thrombotic thrombocytopenic purpura*':ab,ti OR 'thrombotic thrombocytopenic purpura'/exp OR 'moschcowitz disease':ab,ti OR 'moschkowitz disease':ab,ti OR 'upshaw schulman syndrome':ab,ti OR 'upshaw-schulman syndrome'/exp OR 'adamts13 protein':ab,ti OR 'adamts13':ab,ti OR 'adamts 13':ab,ti OR 'von willebrand factor cleaving protease':ab,ti OR 'von willebrand factor cleaving proteinase'/exp OR 'adamts13 protease':ab,ti OR 'vwf cleaving protease':ab,ti | 55,732 |
| Stroke | stroke:ab,ti OR 'cerebrovascular accident'/exp OR 'cerebrovascular accident*':ab,ti OR 'cva':ab,ti OR 'cvas':ab,ti OR 'cerebrovascular apoplexy':ab,ti OR 'brain vascular accident*':ab,ti OR 'ischemic infarct':ab,ti OR 'intracranial hemorrhages':ab,ti OR 'intracranial hemorrhage':ab,ti OR 'brain hemorrhage'/exp OR 'brain hemorrhage*':ab,ti OR 'hemorrhagic transformation':ab,ti OR 'intraparenchymal hemorrhage':ab,ti OR 'hemorrhagic transformation'/exp OR 'intraparenchymal hemorrhage'/exp OR 'microvascular injur*':ab,ti OR 'microvascular injury'/exp OR 'cerebral micro bleed*':ab,ti OR 'neurologic deficit*':ab,ti | 773,709 |
|  |  |  |
| Sum | | |
| **(stroke:ab,ti OR 'cerebrovascular accident'/exp OR 'cerebrovascular accident*':ab,ti OR 'cva':ab,ti OR 'cvas':ab,ti OR 'cerebrovascular apoplexy':ab,ti OR 'brain vascular accident*':ab,ti OR 'ischemic infarct':ab,ti OR 'intracranial hemorrhages':ab,ti OR 'intracranial hemorrhage':ab,ti OR 'brain hemorrhage'/exp OR 'brain hemorrhage*':ab,ti OR 'hemorrhagic transformation':ab,ti OR 'intraparenchymal hemorrhage':ab,ti OR 'hemorrhagic transformation'/exp OR 'intraparenchymal hemorrhage'/exp OR 'microvascular injur*':ab,ti OR 'microvascular injury'/exp OR 'cerebral micro bleed*':ab,ti OR 'neurologic deficit*':ab,ti) AND ('idiopathic thrombocytopenic purpura'/exp OR 'immune thrombocytopenic purpura':ab,ti OR 'immune thrombocytopenia':ab,ti OR 'immune thrombocytopenias':ab,ti OR 'werlhof disease':ab,ti OR 'autoimmune thrombocytopenia':ab,ti OR 'autoimmune thrombocytopenia'/exp OR 'idiopathic thrombocytopenic purpura':ab,ti OR 'idiopathic thrombocytopenic purpuras':ab,ti OR 'thrombotic thrombocytopenic purpura*':ab,ti OR 'thrombotic thrombocytopenic purpura'/exp OR 'moschcowitz disease':ab,ti OR 'moschkowitz disease':ab,ti OR 'upshaw schulman syndrome':ab,ti OR 'upshaw-schulman syndrome'/exp OR 'adamts13 protein':ab,ti OR 'adamts13':ab,ti OR 'adamts 13':ab,ti OR 'von willebrand factor cleaving protease':ab,ti OR 'von willebrand factor cleaving proteinase'/exp OR 'adamts13 protease':ab,ti OR 'vwf cleaving protease':ab,ti)** | | **4,237** |
| Number of Citations after Deduplication |  | **3,664** |

# Cochrane

| Concept | Query | Total |
| --- | --- | --- |
| ITP/TTP | [mh "purpura, thrombocytopenic, idiopathic"] OR "Immune thrombocytopenic purpura":ti,ab OR "Immune Thrombocytopenia":ti,ab OR "Immune Thrombocytopenias":ti,ab OR "Werlhof Disease":ti,ab OR "Autoimmune Thrombocytopenia":ti,ab OR "Autoimmune Thrombocytopenias":ti,ab OR "Idiopathic Thrombocytopenic Purpura":ti,ab OR "Idiopathic Thrombocytopenic Purpuras":ti,ab OR [mh "purpura, thrombotic thrombocytopenic"] OR "thrombotic thrombocytopenic purpura":ti,ab OR "Moschcowitz Disease":ti,ab OR "Moschkowitz Disease":ti,ab OR "Upshaw Schulman Syndrome":ti,ab OR [mh "ADAMTS13 Protein"] OR "ADAMTS13 Protein":ti,ab OR ADAMTS13:ti,ab OR "ADAMTS 13":ti,ab OR "von Willebrand Factor Cleaving Protease":ti,ab OR "ADAMTS13 Protease":ti,ab OR "vWF Cleaving Protease":ti,ab | 1,265 |
| Stroke | [mh "Stroke"] OR "Stroke":ti,ab OR "Strokes":ti,ab OR "cerebrovascular accident":ti,ab OR "cerebrovascular accidents":ti,ab OR "CVA":ti,ab OR "CVAs":ti,ab OR "Cerebrovascular Apoplexy":ti,ab OR "brain vascular accident":ti,ab OR "brain vascular accidents":ti,ab OR "Ischemic Infarct":ti,ab OR [mh "Intracranial Hemorrhages"] OR "Intracranial Hemorrhages":ti,ab OR "Intracranial Hemorrhage":ti,ab OR "brain hemorrhage":ti,ab OR "brain hemorrhages":ti,ab OR "hemorrhagic transformation":ti,ab OR "hemorrhagic transformations":ti,ab OR "intraparenchymal hemorrhage":ti,ab OR "intraparenchymal hemorrhages":ti,ab OR "microvascular injury":ti,ab OR "microvascular injuries":ti,ab OR "cerebral micro bleed":ti,ab OR "cerebral micro bleeding":ti,ab OR "cerebral micro bleeds":ti,ab OR "neurologic deficit":ti,ab OR "neurologic deficits":ti,ab | 69,858 |
|  |  |  |
| Sum | | |
| **([mh "purpura, thrombocytopenic, idiopathic"] OR "Immune thrombocytopenic purpura":ti,ab OR "Immune Thrombocytopenia":ti,ab OR "Immune Thrombocytopenias":ti,ab OR "Werlhof Disease":ti,ab OR "Autoimmune Thrombocytopenia":ti,ab OR "Autoimmune Thrombocytopenias":ti,ab OR "Idiopathic Thrombocytopenic Purpura":ti,ab OR "Idiopathic Thrombocytopenic Purpuras":ti,ab OR [mh "purpura, thrombotic thrombocytopenic"] OR "thrombotic thrombocytopenic purpura":ti,ab OR "Moschcowitz Disease":ti,ab OR "Moschkowitz Disease":ti,ab OR "Upshaw Schulman Syndrome":ti,ab OR [mh "ADAMTS13 Protein"] OR "ADAMTS13 Protein":ti,ab OR ADAMTS13:ti,ab OR "ADAMTS 13":ti,ab OR "von Willebrand Factor Cleaving Protease":ti,ab OR "ADAMTS13 Protease":ti,ab OR "vWF Cleaving Protease":ti,ab) AND ([mh "Stroke"] OR "Stroke":ti,ab OR "Strokes":ti,ab OR "cerebrovascular accident":ti,ab OR "cerebrovascular accidents":ti,ab OR "CVA":ti,ab OR "CVAs":ti,ab OR "Cerebrovascular Apoplexy":ti,ab OR "brain vascular accident":ti,ab OR "brain vascular accidents":ti,ab OR "Ischemic Infarct":ti,ab OR [mh "Intracranial Hemorrhages"] OR "Intracranial Hemorrhages":ti,ab OR "Intracranial Hemorrhage":ti,ab OR "brain hemorrhage":ti,ab OR "brain hemorrhages":ti,ab OR "hemorrhagic transformation":ti,ab OR "hemorrhagic transformations":ti,ab OR "intraparenchymal hemorrhage":ti,ab OR "intraparenchymal hemorrhages":ti,ab OR "microvascular injury":ti,ab OR "microvascular injuries":ti,ab OR "cerebral micro bleed":ti,ab OR "cerebral micro bleeding":ti,ab OR "cerebral micro bleeds":ti,ab OR "neurologic deficit":ti,ab OR "neurologic deficits":ti,ab)** | | **36** |
| Number of Citations after Deduplication |  | **14** |

# Web of Science

| Concept | Query | Total |
| --- | --- | --- |
| ITP/TTP | TS=("Immune thrombocytopenic purpura*" OR "Immune Thrombocytopenia" OR "Immune Thrombocytopenias" OR "Werlhof Disease" OR "Autoimmune Thrombocytopenia" OR "Autoimmune Thrombocytopenias" OR "Idiopathic Thrombocytopenic Purpura" OR "Idiopathic Thrombocytopenic Purpuras" OR "thrombotic thrombocytopenic purpura" OR "Moschcowitz Disease" OR "Moschkowitz Disease" OR "Upshaw Schulman Syndrome" OR "ADAMTS13 Protein" OR "ADAMTS13 Protein" OR ADAMTS13 OR "ADAMTS 13" OR "von Willebrand Factor Cleaving Protease" OR "ADAMTS13 Protease" OR "vWF Cleaving Protease") | 24,218 |
| Stroke | TS=(Stroke OR Stroke OR Strokes OR "cerebrovascular accident*" OR CVA OR CVAs OR "Cerebrovascular Apoplexy" OR "brain vascular accident*" OR "Ischemic Infarct" OR ("Intracranial Hemorrhages" OR "Intracranial Hemorrhages" OR "Intracranial Hemorrhage" OR "brain hemorrhage*") OR ("hemorrhagic transformation*" OR "intraparenchymal hemorrhage*" OR "microvascular injur*" OR "cerebral micro bleed*" OR "neurologic deficit*")) | 489,191 |
|  |  |  |
| Sum | | |
| **(TS=(“Immune thrombocytopenic purpura*" OR "Immune Thrombocytopenia" OR "Immune Thrombocytopenias" OR "Werlhof Disease" OR "Autoimmune Thrombocytopenia" OR "Autoimmune Thrombocytopenias" OR "Idiopathic Thrombocytopenic Purpura" OR "Idiopathic Thrombocytopenic Purpuras" OR "thrombotic thrombocytopenic purpura*" OR "Moschcowitz Disease" OR "Moschkowitz Disease" OR "Upshaw Schulman Syndrome" OR "ADAMTS13 Protein" OR "ADAMTS13 Protein" OR ADAMTS13 OR "ADAMTS 13" OR "von Willebrand Factor Cleaving Protease" OR "ADAMTS13 Protease" OR "vWF Cleaving Protease")) AND (TS=(Stroke OR Stroke OR Strokes OR "cerebrovascular accident*" OR CVA OR CVAs OR "Cerebrovascular Apoplexy" OR "brain vascular accident*" OR "Ischemic Infarct" OR ("Intracranial Hemorrhages" OR "Intracranial Hemorrhages" OR "Intracranial Hemorrhage" OR "brain hemorrhage*") OR ("hemorrhagic transformation*" OR "intraparenchymal hemorrhage*" OR "microvascular injur*" OR "cerebral micro bleed*" OR "neurologic deficit*")))** | | **822** |
| Number of Citations after Deduplication |  | **396** |

# Scopus

| Concept | Query | | Total |
| --- | --- | --- | --- |
| ITP/TTP | TITLE-ABS-KEY ( "Immune thrombocytopenic purpura" OR "Immune Thrombocytopenia" OR "Immune Thrombocytopenias" OR "Werlhof Disease" OR "Autoimmune Thrombocytopenia" OR "Autoimmune Thrombocytopenias" OR "Idiopathic Thrombocytopenic Purpura" OR "Idiopathic Thrombocytopenic Purpuras" OR "thrombotic thrombocytopenic purpura*" OR "Moschcowitz Disease" OR "Moschkowitz Disease" OR "Upshaw Schulman Syndrome" OR "ADAMTS13 Protein" OR "ADAMTS13 Protein" OR adamts13 OR "ADAMTS 13" OR "von Willebrand Factor Cleaving Protease" OR "ADAMTS13 Protease" OR "vWF Cleaving Protease" ) | | 35,942 |
| Stroke | TITLE-ABS-KEY ( stroke OR strokes OR "cerebrovascular accident*" OR cva OR cvas OR "Cerebrovascular Apoplexy" OR "brain vascular accident*" OR "Ischemic Infarct" OR ( "Intracranial Hemorrhages" OR "Intracranial Hemorrhages" OR "Intracranial Hemorrhage" OR "brain hemorrhage*" ) OR ( "hemorrhagic transformation*" OR "intraparenchymal hemorrhage*" OR "microvascular injur*" OR "cerebral micro bleed*" OR "neurologic deficit*" ) ) | | 692,040 |
|  |  | |  |
| Sum | | | |
| **( TITLE-ABS-KEY ( "Immune thrombocytopenic purpura" OR "Immune Thrombocytopenia" OR "Immune Thrombocytopenias" OR "Werlhof Disease" OR "Autoimmune Thrombocytopenia" OR "Autoimmune Thrombocytopenias" OR "Idiopathic Thrombocytopenic Purpura" OR "Idiopathic Thrombocytopenic Purpuras" OR "thrombotic thrombocytopenic purpura*" OR "Moschcowitz Disease" OR "Moschkowitz Disease" OR "Upshaw Schulman Syndrome" OR "ADAMTS13 Protein" OR "ADAMTS13 Protein" OR adamts13 OR "ADAMTS 13" OR "von Willebrand Factor Cleaving Protease" OR "ADAMTS13 Protease" OR "vWF Cleaving Protease" ) ) AND ( TITLE-ABS-KEY ( stroke OR strokes OR "cerebrovascular accident*" OR cva OR cvas OR "Cerebrovascular Apoplexy" OR "brain vascular accident*" OR "Ischemic Infarct" OR ( "Intracranial Hemorrhages" OR "Intracranial Hemorrhages" OR "Intracranial Hemorrhage" OR "brain hemorrhage*" ) OR ( "hemorrhagic transformation*" OR "intraparenchymal hemorrhage*" OR "microvascular injur*" OR "cerebral micro bleed*" OR "neurologic deficit*" ) ) )** | | | **2,327** |
| Number of Citations after Deduplication | |  | **229** |
|  |  |  |  |

# Citation Manager - EndNote

| **Database** | **Number of Citations Before Deduplication** | **Number of Citations After Deduplication** |
| --- | --- | --- |
| PubMed | 834 | 834 |
| Embase | 4,237 | 3,664 |
| Cochrane | 36 | 14 |
| Web of Science | 822 | 396 |
| SCOPUS | 2,327 | 229 |
| **Total** | **8,256** | **5,137** |

**Appendix 2: NOS Bias Assessment for included studies.**

| **Author Name** | **Population** | **Study Design** | **Setting** | **Selection** | **Comparability** | **Outcome** | **Total** |
| --- | --- | --- | --- | --- | --- | --- | --- |
| Adeyemi, 2022 | 666 | Longitudinal, retrospective observational study | Multicenter | **** | ** | *** | 9 |
| Agosti, 2020 | 36 | Retrospective case control | Multicenter | **** | ** | *** | 7 |
| Altomare, 2016 | 6651 | Retrospective cohort study | Multicenter | **** | ** | *** | 9 |
| Arnold, 2017 | 259 | Prospective longitudinal | Single center | **** | ** | *** | 9 |
| Brodsky, 2021 | 181 | Retrospective cohort study | Multicenter | **** | ** | *** | 9 |
| Chaturvedi, 2023 | 42 | Prospective cohort | Single center | **** | ** | *** | 9 |
| Chehab, 2019 | 1002 | Retrospective propensity matched cohort | Multicenter | **** | ** | *** | 9 |
| Chen, 2022 | 13085 | Retrospective chart review | Multicenter | **** | ** | *** | 9 |
| Chong, 2022 | 142 | Retrospective cohort | Multicenter | **** | ** | *** | 9 |
| Cooper, 2020 | 49 | Retrospective cohort | Single center | *** | ** | *** | 9 |
| Hallan, 2022 | 58835 | Case control | Multicenter | *** | ** | *** | 8 |
| Hamzah, 2022 | 500 | Retrospective cohort | Multicenter | **** | * | *** | 9 |
| Hato, 2020 | 19415 | Retrospective cohort | Multicenter | **** | ** | *** | 9 |
| Kim, 2013 | 165 | Retrospective cohort | Single center | **** | ** | *** | 9 |
| Kueh, 1995 | 37 | Prospective cohort | Single center | ** |  | *** | 5 |
| Kuhne, 2011 | 340 | Retrospective cohort | Multicenter | **** |  | *** | 8 |
| Lal, 2020 | 3332 | Retrospective observational | Multicenter | **** |  | *** | 8 |
| Li, 2018 | 3007 | Retrospective observational | Multicenter | **** |  |  | 7 |
| Lin, 2021 | 108 | Retrospective observational | Multicenter | **** | * | *** | 8 |
| Lopez, 2020 | 145 | Retrospective cohort | Multicenter | **** |  | *** | 7 |
| Lopez, 2017 | 220 | Retrospective cohort | Multicenter | *** | * | *** | 7 |
| Mazza, 2016 | 124 | Retrospective observational | Multicenter | *** | ** | *** | 8 |
| Memon, 2021 | 66 | Retrospective observational | Multicenter | *** | ** | *** | 9 |
| Michel, 2011 | 152 | Case-control retrospective study | Single center | **** | * | ** | 7 |
| Mirouse, 2021 | 108 | Prospective cohort | Single center | *** | ** | *** | 8 |
| Moulis, 2017 | 113 | Prospective cohort | Multicenter | **** | ** | *** | 9 |
| Neuman, 2023 | 57 | Prospective multicenter study | Multicenter | **** | ** | *** | 9 |
| Nichols, 2015 | 18 | Retrospective observational | Single center | *** |  | *** | 6 |
| Nørgaard, 2011 | 4476 | Case control | Multicenter | **** | ** | *** | 9 |
| Palandri, 2020 | 451 | Retrospective observational | Multicenter | *** | ** | *** | 8 |
| Panicker, 2008 | 7 | Case series | Single center | ** |  | *** | 5 |
| Park, 2014 | 2185 | Retrospective observational | Single center | **** | * | *** | 7 |
| Piel-Julian, 2018 | 302 | Cross sectional | Multicenter | **** | ** | *** | 9 |
| Rio-Garma, 2022 | 102 | Retrospective cohort | Multicenter | **** | ** | *** | 9 |
| Rong, 2022 | 1824 | Retrospective cohort | Single center | **** | ** | *** | 9 |
| Rosove, 1982 | 19 | Case series | Multicenter | ** |  | ** | 4 |
| Sakai, 2021 | 55 | Retrospective cohort | Multicenter | **** |  | *** | 7 |
| Schattner, 1994 | 6 | Case series | Single center | ** |  | *** | 5 |
| Szende, 2010 | 9 | Literature review | N/A | **** | ** | *** | 9 |
| Tsuda, 2017 | 132 | Retrospective cohort | Single center | **** | ** | *** | 9 |
| Upreti, 2019 | 137 | Retrospective review | Single center | **** | ** | *** | 9 |
| Wong, 2023 | 51 | Retrospective-prospective cohort study | Single center | **** |  | *** | 7 |
| Zhao, 2020 | 279 | Retrospective case-control | Multicenter | **** | ** | *** | 9 |
| Zhou, 2015 | 15 | Retrospective case series | Single center | ** |  | *** | 5 |
| **9 [7-9]** | | | | | | | |

**Appendix 3: The revised Cochrane risk-of-bias tool for randomized trials (RoB 2)**

| **Study details**   \| **Reference** \| Evaluation of bleeding and thrombotic events during long-term use of romiplostim in patients with chronic immune thrombocytopenia (ITP) \| \| --- \| --- \|   **Study design**   \| X \| Individually-randomized parallel-group trial \| \| --- \| --- \| \| ⬜ \| Cluster-randomized parallel-group trial \| \| ⬜ \| Individually randomized cross-over (or other matched) trial \|   **For the purposes of this assessment, the interventions being compared are defined as**   \| Experimental: \| Romiplostim \| Comparator: \| No romiplostim \| \| --- \| --- \| --- \| --- \|  \| **Specify which outcome is being assessed for risk of bias** \| Bleeding and thrombotic events \| \| --- \| --- \|  \| **Specify the numerical result being assessed.** In case of multiple alternative analyses being presented, specify the numeric result (e.g. RR = 1.52 (95% CI 0.83 to 2.77) and/or a reference (e.g. to a table, figure or paragraph) that uniquely defines the result being assessed. \| Tables 2-5 \| \| --- \| --- \|   **Is the review team’s aim for this result…?**   \| X \| to assess the effect of *assignment to intervention* (the ‘intention-to-treat’ effect) \| \| --- \| --- \| \| ⬜ \| to assess the effect of *adhering to intervention* (the ‘per-protocol’ effect) \|   **If the aim is to assess the effect of *adhering to intervention***, select the deviations from intended intervention that should be addressed (at least one must be checked):  ⬜ occurrence of non-protocol interventions  ⬜ failures in implementing the intervention that could have affected the outcome  ⬜ non-adherence to their assigned intervention by trial participants  **Which of the following sources were obtained to help inform the risk-of-bias assessment? (tick as many as apply)**  X Journal article(s) with results of the trial  ⬜ Trial protocol  ⬜ Statistical analysis plan (SAP)  ⬜ Non-commercial trial registry record (e.g. ClinicalTrials.gov record)  ⬜ Company-owned trial registry record (e.g. GSK Clinical Study Register record)  ⬜ “Grey literature” (e.g. unpublished thesis)  ⬜ Conference abstract(s) about the trial  ⬜ Regulatory document (e.g. Clinical Study Report, Drug Approval Package)  ⬜ Research ethics application  ⬜ Grant database summary (e.g. NIH RePORTER or Research Councils UK Gateway to Research)  ⬜ Personal communication with trialist  ⬜ Personal communication with the sponsor |
| --- | --- | --- | --- | --- | --- | --- | --- | --- | --- | --- | --- | --- | --- | --- | --- | --- | --- | --- | --- | --- |

## Risk of bias assessment

Responses underlined in green are potential markers for low risk of bias, and responses in red are potential markers for a risk of bias. Where questions relate only to sign posts to other questions, no formatting is used.

**Domain 1: Risk of bias arising from the randomization process**

| **Signalling questions** | **Comments** | **Response options** |
| --- | --- | --- |
| **1.1 Was the allocation sequence random?** |  | Y |
| **1.2 Was the allocation sequence concealed until participants were enrolled and assigned to interventions?** |  | Y |
| **1.3 Did baseline differences between intervention groups suggest a problem with the randomization process?** |  | N |
| **Risk-of-bias judgement** |  | Low |
| Optional: What is the predicted direction of bias arising from the randomization process? |  | NA |

Domain 2: Risk of bias due to deviations from the intended interventions (*effect of assignment to intervention*)

| **Signalling questions** | **Comments** | **Response options** |
| --- | --- | --- |
| **2.1. Were participants aware of their assigned intervention during the trial?** |  | N |
| **2.2. Were carers and people delivering the interventions aware of participants' assigned intervention during the trial?** |  | N |
| **2.3. If Y/PY/NI to 2.1 or 2.2: Were there deviations from the intended intervention that arose because of the trial context?** |  | NA |
| **2.4 If Y/PY to 2.3: Were these deviations likely to have affected the outcome?** |  | NA |
| **2.5. If Y/PY/NI to 2.4: Were these deviations from intended intervention balanced between groups?** |  | NA |
| **2.6 Was an appropriate analysis used to estimate the effect of assignment to intervention?** |  | Y |
| **2.7 If N/PN/NI to 2.6: Was there potential for a substantial impact (on the result) of the failure to analyse participants in the group to which they were randomized?** |  | NA |
| **Risk-of-bias judgement** |  | Low |
| Optional: What is the predicted direction of bias due to deviations from intended interventions? |  | NA |

Domain 2: Risk of bias due to deviations from the intended interventions (*effect of adhering to intervention*)

| **Signalling questions** | **Comments** | **Response options** |
| --- | --- | --- |
| **2.1. Were participants aware of their assigned intervention during the trial?** |  | N |
| **2.2. Were carers and people delivering the interventions aware of participants' assigned intervention during the trial?** |  | N |
| **2.3. [If applicable:] If Y/PY/NI to 2.1 or 2.2: Were important non-protocol interventions balanced across intervention groups?** |  | NA |
| **2.4. [If applicable:] Were there failures in implementing the intervention that could have affected the outcome?** |  | N |
| **2.5. [If applicable:] Was there non-adherence to the assigned intervention regimen that could have affected participants’ outcomes?** |  | N |
| **2.6. If N/PN/NI to 2.3, or Y/PY/NI to 2.4 or 2.5: Was an appropriate analysis used to estimate the effect of adhering to the intervention?** |  | NA |
| **Risk-of-bias judgement** |  | Low |
| Optional: What is the predicted direction of bias due to deviations from intended interventions? |  | NA |

Domain 3: Missing outcome data

| **Signalling questions** | **Comments** | **Response options** |
| --- | --- | --- |
| **3.1 Were data for this outcome available for all, or nearly all, participants randomized?** |  | Y |
| **3.2 If N/PN/NI to 3.1: Is there evidence that the result was not biased by missing outcome data?** |  | NA |
| **3.3 If N/PN to 3.2: Could missingness in the outcome depend on its true value?** |  | NA |
| **3.4 If Y/PY/NI to 3.3: Is it likely that missingness in the outcome depended on its true value?** |  | NA |
| **Risk-of-bias judgement** |  | Low |
| Optional: What is the predicted direction of bias due to missing outcome data? |  | NA |

Domain 4: Risk of bias in measurement of the outcome

| **Signalling questions** | **Comments** | **Response options** |
| --- | --- | --- |
| **4.1 Was the method of measuring the outcome inappropriate?** |  | N |
| **4.2 Could measurement or ascertainment of the outcome have differed between intervention groups?** |  | N |
| **4.3 If N/PN/NI to 4.1 and 4.2: Were outcome assessors aware of the intervention received by study participants?** |  | NI |
| **4.4 If Y/PY/NI to 4.3: Could assessment of the outcome have been influenced by knowledge of intervention received?** |  | PN |
| **4.5 If Y/PY/NI to 4.4: Is it likely that assessment of the outcome was influenced by knowledge of intervention received?** |  | NA |
| **Risk-of-bias judgement** |  | Low |
| Optional: What is the predicted direction of bias in measurement of the outcome? |  | NA |

Domain 5: Risk of bias in selection of the reported result

| **Signalling questions** | **Comments** | **Response options** |
| --- | --- | --- |
| **5.1 Were the data that produced this result analysed in accordance with a pre-specified analysis plan that was finalized before unblinded outcome data were available for analysis?** |  | Y |
| **Is the numerical result being assessed likely to have been selected, on the basis of the results, from...** |  |  |
| **5.2. ... multiple eligible outcome measurements (e.g. scales, definitions, time points) within the outcome domain?** |  | N |
| **5.3 ... multiple eligible analyses of the data?** |  | N |
| **Risk-of-bias judgement** |  | Low |
| Optional: What is the predicted direction of bias due to selection of the reported result? |  | NA |

Overall risk of bias

| **Risk-of-bias judgement** |  | Low |
| --- | --- | --- |
| Optional: What is the overall predicted direction of bias for this outcome? |  | NA |


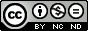


This work is licensed under a [Creative Commons Attribution-NonCommercial-NoDerivatives 4.0 International License](http://creativecommons.org/licenses/by-nc-nd/4.0/).

Page break

Appendix: The revised Cochrane risk-of-bias tool for randomized trials (RoB 2)

| **Study details**   \| **Reference** \| A multicenter randomized controlled trial of recombinant human thrombopoietin treatment in patients with primary immune thrombocytopenia \| \| --- \| --- \|   **Study design**   \| X \| Individually-randomized parallel-group trial \| \| --- \| --- \| \| ⬜ \| Cluster-randomized parallel-group trial \| \| ⬜ \| Individually randomized cross-over (or other matched) trial \|   **For the purposes of this assessment, the interventions being compared are defined as**   \| Experimental: \| rhTPO + danazol \| Comparator: \| danazol \| \| --- \| --- \| --- \| --- \|  \| **Specify which outcome is being assessed for risk of bias** \| Changes in platelet counts and efficacy during the first phase \| \| --- \| --- \|  \| **Specify the numerical result being assessed.** In case of multiple alternative analyses being presented, specify the numeric result (e.g. RR = 1.52 (95% CI 0.83 to 2.77) and/or a reference (e.g. to a table, figure or paragraph) that uniquely defines the result being assessed. \| Table 2 \| \| --- \| --- \|   **Is the review team’s aim for this result…?**   \| X \| to assess the effect of *assignment to intervention* (the ‘intention-to-treat’ effect) \| \| --- \| --- \| \| ⬜ \| to assess the effect of *adhering to intervention* (the ‘per-protocol’ effect) \|   **If the aim is to assess the effect of *adhering to intervention***, select the deviations from intended intervention that should be addressed (at least one must be checked):  ⬜ occurrence of non-protocol interventions  ⬜ failures in implementing the intervention that could have affected the outcome  ⬜ non-adherence to their assigned intervention by trial participants  **Which of the following sources were obtained to help inform the risk-of-bias assessment? (tick as many as apply)**  X Journal article(s) with results of the trial  ⬜ Trial protocol  ⬜ Statistical analysis plan (SAP)  ⬜ Non-commercial trial registry record (e.g. ClinicalTrials.gov record)  ⬜ Company-owned trial registry record (e.g. GSK Clinical Study Register record)  ⬜ “Grey literature” (e.g. unpublished thesis)  ⬜ Conference abstract(s) about the trial  ⬜ Regulatory document (e.g. Clinical Study Report, Drug Approval Package)  ⬜ Research ethics application  ⬜ Grant database summary (e.g. NIH RePORTER or Research Councils UK Gateway to Research)  ⬜ Personal communication with trialist  ⬜ Personal communication with the sponsor |
| --- | --- | --- | --- | --- | --- | --- | --- | --- | --- | --- | --- | --- | --- | --- | --- | --- | --- | --- | --- | --- |

## Risk of bias assessment

Responses underlined in green are potential markers for low risk of bias, and responses in red are potential markers for a risk of bias. Where questions relate only to sign posts to other questions, no formatting is used.

**Domain 1: Risk of bias arising from the randomization process**

| **Signalling questions** | **Comments** | **Response options** |
| --- | --- | --- |
| **1.1 Was the allocation sequence random?** |  | Y |
| **1.2 Was the allocation sequence concealed until participants were enrolled and assigned to interventions?** |  | Y |
| **1.3 Did baseline differences between intervention groups suggest a problem with the randomization process?** |  | N |
| **Risk-of-bias judgement** |  | Low |
| Optional: What is the predicted direction of bias arising from the randomization process? |  | NA |

Domain 2: Risk of bias due to deviations from the intended interventions (*effect of assignment to intervention*)

| **Signalling questions** | **Comments** | **Response options** |
| --- | --- | --- |
| **2.1. Were participants aware of their assigned intervention during the trial?** |  | NI |
| **2.2. Were carers and people delivering the interventions aware of participants' assigned intervention during the trial?** |  | NI |
| **2.3. If Y/PY/NI to 2.1 or 2.2: Were there deviations from the intended intervention that arose because of the trial context?** |  | N |
| **2.4 If Y/PY to 2.3: Were these deviations likely to have affected the outcome?** |  | NA |
| **2.5. If Y/PY/NI to 2.4: Were these deviations from intended intervention balanced between groups?** |  | NA |
| **2.6 Was an appropriate analysis used to estimate the effect of assignment to intervention?** |  | Y |
| **2.7 If N/PN/NI to 2.6: Was there potential for a substantial impact (on the result) of the failure to analyse participants in the group to which they were randomized?** |  | NA |
| **Risk-of-bias judgement** |  | Some concerns |
| Optional: What is the predicted direction of bias due to deviations from intended interventions? |  | NA |

Domain 2: Risk of bias due to deviations from the intended interventions (*effect of adhering to intervention*)

| **Signalling questions** | **Comments** | **Response options** |
| --- | --- | --- |
| **2.1. Were participants aware of their assigned intervention during the trial?** |  | NI |
| **2.2. Were carers and people delivering the interventions aware of participants' assigned intervention during the trial?** |  | NI |
| **2.3. [If applicable:] If Y/PY/NI to 2.1 or 2.2: Were important non-protocol interventions balanced across intervention groups?** |  | N |
| **2.4. [If applicable:] Were there failures in implementing the intervention that could have affected the outcome?** |  | N |
| **2.5. [If applicable:] Was there non-adherence to the assigned intervention regimen that could have affected participants’ outcomes?** |  | N |
| **2.6. If N/PN/NI to 2.3, or Y/PY/NI to 2.4 or 2.5: Was an appropriate analysis used to estimate the effect of adhering to the intervention?** |  | NA |
| **Risk-of-bias judgement** |  | Some ccncerns |
| Optional: What is the predicted direction of bias due to deviations from intended interventions? |  | NA |

Domain 3: Missing outcome data

| **Signalling questions** | **Comments** | **Response options** |
| --- | --- | --- |
| **3.1 Were data for this outcome available for all, or nearly all, participants randomized?** |  | Y |
| **3.2 If N/PN/NI to 3.1: Is there evidence that the result was not biased by missing outcome data?** |  | NA |
| **3.3 If N/PN to 3.2: Could missingness in the outcome depend on its true value?** |  | NA |
| **3.4 If Y/PY/NI to 3.3: Is it likely that missingness in the outcome depended on its true value?** |  | NA |
| **Risk-of-bias judgement** |  | Low |
| Optional: What is the predicted direction of bias due to missing outcome data? |  | NA |

Domain 4: Risk of bias in measurement of the outcome

| **Signalling questions** | **Comments** | **Response options** |
| --- | --- | --- |
| **4.1 Was the method of measuring the outcome inappropriate?** |  | N |
| **4.2 Could measurement or ascertainment of the outcome have differed between intervention groups?** |  | N |
| **4.3 If N/PN/NI to 4.1 and 4.2: Were outcome assessors aware of the intervention received by study participants?** |  | NI |
| **4.4 If Y/PY/NI to 4.3: Could assessment of the outcome have been influenced by knowledge of intervention received?** |  | PN |
| **4.5 If Y/PY/NI to 4.4: Is it likely that assessment of the outcome was influenced by knowledge of intervention received?** |  | NA |
| **Risk-of-bias judgement** |  | Low |
| Optional: What is the predicted direction of bias in measurement of the outcome? |  | NA / Favours experimental / Favours comparator / Towards null /Away from null / Unpredictable |

Domain 5: Risk of bias in selection of the reported result

| **Signalling questions** | **Comments** | **Response options** |
| --- | --- | --- |
| **5.1 Were the data that produced this result analysed in accordance with a pre-specified analysis plan that was finalized before unblinded outcome data were available for analysis?** |  | Y |
| **Is the numerical result being assessed likely to have been selected, on the basis of the results, from...** |  |  |
| **5.2. ... multiple eligible outcome measurements (e.g. scales, definitions, time points) within the outcome domain?** |  | N |
| **5.3 ... multiple eligible analyses of the data?** |  | N |
| **Risk-of-bias judgement** |  | Low |
| Optional: What is the predicted direction of bias due to selection of the reported result? |  | NA |

Overall risk of bias

| **Risk-of-bias judgement** |  | Low |
| --- | --- | --- |
| Optional: What is the overall predicted direction of bias for this outcome? |  | NA |


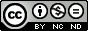


This work is licensed under a [Creative Commons Attribution-NonCommercial-NoDerivatives 4.0 International License](http://creativecommons.org/licenses/by-nc-nd/4.0/).

Appendix: The revised Cochrane risk-of-bias tool for randomized trials (RoB 2)

| **Study details**   \| **Reference** \| The Outcomes and Adverse Drug Patterns of Immunomodulators and Thrombopoietin Receptor Agonists in Primary Immune Thrombocytopenia Egyptian Patients with Hemorrhage comorbidity \| \| --- \| --- \|   **Study design**   \| X \| Individually-randomized parallel-group trial \| \| --- \| --- \| \| ⬜ \| Cluster-randomized parallel-group trial \| \| ⬜ \| Individually randomized cross-over (or other matched) trial \|   **For the purposes of this assessment, the interventions being compared are defined as**   \| Experimental: \| Eltrombopag,Romiplostim,Prednisolone+Azathioprine,Rituximab \| Comparator: \| High Dose-dexamethasone \| \| --- \| --- \| --- \| --- \|  \| **Specify which outcome is being assessed for risk of bias** \| Total percentage of patients achieving a sustained response, defined as sustaining PLTs counts over 50 × 109/L for an additional 6 months without extra ITP regimens. \| \| --- \| --- \|  \| **Specify the numerical result being assessed.** In case of multiple alternative analyses being presented, specify the numeric result (e.g. RR = 1.52 (95% CI 0.83 to 2.77) and/or a reference (e.g. to a table, figure or paragraph) that uniquely defines the result being assessed. \| Table 2 \| \| --- \| --- \|   **Is the review team’s aim for this result…?**   \| X \| to assess the effect of *assignment to intervention* (the ‘intention-to-treat’ effect) \| \| --- \| --- \| \| ⬜ \| to assess the effect of *adhering to intervention* (the ‘per-protocol’ effect) \|   **If the aim is to assess the effect of *adhering to intervention***, select the deviations from intended intervention that should be addressed (at least one must be checked):  ⬜ occurrence of non-protocol interventions  ⬜ failures in implementing the intervention that could have affected the outcome  ⬜ non-adherence to their assigned intervention by trial participants  **Which of the following sources were obtained to help inform the risk-of-bias assessment? (tick as many as apply)**  X Journal article(s) with results of the trial  ⬜ Trial protocol  ⬜ Statistical analysis plan (SAP)  ⬜ Non-commercial trial registry record (e.g. ClinicalTrials.gov record)  ⬜ Company-owned trial registry record (e.g. GSK Clinical Study Register record)  ⬜ “Grey literature” (e.g. unpublished thesis)  ⬜ Conference abstract(s) about the trial  ⬜ Regulatory document (e.g. Clinical Study Report, Drug Approval Package)  ⬜ Research ethics application  ⬜ Grant database summary (e.g. NIH RePORTER or Research Councils UK Gateway to Research)  ⬜ Personal communication with trialist  ⬜ Personal communication with the sponsor |
| --- | --- | --- | --- | --- | --- | --- | --- | --- | --- | --- | --- | --- | --- | --- | --- | --- | --- | --- | --- | --- |

## Risk of bias assessment

Responses underlined in green are potential markers for low risk of bias, and responses in red are potential markers for a risk of bias. Where questions relate only to sign posts to other questions, no formatting is used.

**Domain 1: Risk of bias arising from the randomization process**

| **Signalling questions** | **Comments** | **Response options** |
| --- | --- | --- |
| **1.1 Was the allocation sequence random?** |  | Y |
| **1.2 Was the allocation sequence concealed until participants were enrolled and assigned to interventions?** |  | Y |
| **1.3 Did baseline differences between intervention groups suggest a problem with the randomization process?** |  | N |
| **Risk-of-bias judgement** |  | Low |
| Optional: What is the predicted direction of bias arising from the randomization process? |  | NA |

Domain 2: Risk of bias due to deviations from the intended interventions (*effect of assignment to intervention*)

| **Signalling questions** | **Comments** | **Response options** |
| --- | --- | --- |
| **2.1. Were participants aware of their assigned intervention during the trial?** |  | NI |
| **2.2. Were carers and people delivering the interventions aware of participants' assigned intervention during the trial?** |  | NI |
| **2.3. If Y/PY/NI to 2.1 or 2.2: Were there deviations from the intended intervention that arose because of the trial context?** |  | N |
| **2.4 If Y/PY to 2.3: Were these deviations likely to have affected the outcome?** |  | NA |
| **2.5. If Y/PY/NI to 2.4: Were these deviations from intended intervention balanced between groups?** |  | NA |
| **2.6 Was an appropriate analysis used to estimate the effect of assignment to intervention?** |  | Y |
| **2.7 If N/PN/NI to 2.6: Was there potential for a substantial impact (on the result) of the failure to analyse participants in the group to which they were randomized?** |  | NA |
| **Risk-of-bias judgement** |  | Some concerns |
| Optional: What is the predicted direction of bias due to deviations from intended interventions? |  | NA |

Domain 2: Risk of bias due to deviations from the intended interventions (*effect of adhering to intervention*)

| **Signalling questions** | **Comments** | **Response options** |
| --- | --- | --- |
| **2.1. Were participants aware of their assigned intervention during the trial?** |  | NI |
| **2.2. Were carers and people delivering the interventions aware of participants' assigned intervention during the trial?** |  | NI |
| **2.3. [If applicable:] If Y/PY/NI to 2.1 or 2.2: Were important non-protocol interventions balanced across intervention groups?** |  | Y |
| **2.4. [If applicable:] Were there failures in implementing the intervention that could have affected the outcome?** |  | N |
| **2.5. [If applicable:] Was there non-adherence to the assigned intervention regimen that could have affected participants’ outcomes?** |  | N |
| **2.6. If N/PN/NI to 2.3, or Y/PY/NI to 2.4 or 2.5: Was an appropriate analysis used to estimate the effect of adhering to the intervention?** |  | NA |
| **Risk-of-bias judgement** |  | Some concerns |
| Optional: What is the predicted direction of bias due to deviations from intended interventions? |  | NA |

Domain 3: Missing outcome data

| **Signalling questions** | **Comments** | **Response options** |
| --- | --- | --- |
| **3.1 Were data for this outcome available for all, or nearly all, participants randomized?** |  | Y |
| **3.2 If N/PN/NI to 3.1: Is there evidence that the result was not biased by missing outcome data?** |  | NA |
| **3.3 If N/PN to 3.2: Could missingness in the outcome depend on its true value?** |  | NA |
| **3.4 If Y/PY/NI to 3.3: Is it likely that missingness in the outcome depended on its true value?** |  | NA |
| **Risk-of-bias judgement** |  | Low |
| Optional: What is the predicted direction of bias due to missing outcome data? |  | NA |

Domain 4: Risk of bias in measurement of the outcome

| **Signalling questions** | **Comments** | **Response options** |
| --- | --- | --- |
| **4.1 Was the method of measuring the outcome inappropriate?** |  | N |
| **4.2 Could measurement or ascertainment of the outcome have differed between intervention groups?** |  | N |
| **4.3 If N/PN/NI to 4.1 and 4.2: Were outcome assessors aware of the intervention received by study participants?** |  | NI |
| **4.4 If Y/PY/NI to 4.3: Could assessment of the outcome have been influenced by knowledge of intervention received?** |  | U |
| **4.5 If Y/PY/NI to 4.4: Is it likely that assessment of the outcome was influenced by knowledge of intervention received?** |  | NA |
| **Risk-of-bias judgement** |  | Low |
| Optional: What is the predicted direction of bias in measurement of the outcome? |  | NA / Favours experimental / Favours comparator / Towards null /Away from null / Unpredictable |

Domain 5: Risk of bias in selection of the reported result

| **Signalling questions** | **Comments** | **Response options** |
| --- | --- | --- |
| **5.1 Were the data that produced this result analysed in accordance with a pre-specified analysis plan that was finalized before unblinded outcome data were available for analysis?** |  | Y |
| **Is the numerical result being assessed likely to have been selected, on the basis of the results, from...** |  |  |
| **5.2. ... multiple eligible outcome measurements (e.g. scales, definitions, time points) within the outcome domain?** |  | N |
| **5.3 ... multiple eligible analyses of the data?** |  | N |
| **Risk-of-bias judgement** |  | Low |
| Optional: What is the predicted direction of bias due to selection of the reported result? |  | NA |

Overall risk of bias

| **Risk-of-bias judgement** |  | Low |
| --- | --- | --- |
| Optional: What is the overall predicted direction of bias for this outcome? |  | NA |


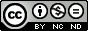


This work is licensed under a [Creative Commons Attribution-NonCommercial-NoDerivatives 4.0 International License](http://creativecommons.org/licenses/by-nc-nd/4.0/).

Appendix: The revised Cochrane risk-of-bias tool for randomized trials (RoB 2)

| **Study details**   \| **Reference** \| Caplacizumab reduces the frequency of major thromboembolic events, exacerbations and death in patients with acquired thrombotic thrombocytopenic purpura \| \| --- \| --- \|   **Study design**   \| X \| Individually-randomized parallel-group trial \| \| --- \| --- \| \| ⬜ \| Cluster-randomized parallel-group trial \| \| ⬜ \| Individually randomized cross-over (or other matched) trial \|   **For the purposes of this assessment, the interventions being compared are defined as**   \| Experimental: \| Caplacizumab (10 mg) \| Comparator: \| Placebo \| \| --- \| --- \| --- \| --- \|  \| **Specify which outcome is being assessed for risk of bias** \| Incidence of major thromboembolic events, aTTP exacerbations, and aTTP-related mortality \| \| --- \| --- \|  \| **Specify the numerical result being assessed.** In case of multiple alternative analyses being presented, specify the numeric result (e.g. RR = 1.52 (95% CI 0.83 to 2.77) and/or a reference (e.g. to a table, figure or paragraph) that uniquely defines the result being assessed. \| Table 1 \| \| --- \| --- \|   **Is the review team’s aim for this result…?**   \| X \| to assess the effect of *assignment to intervention* (the ‘intention-to-treat’ effect) \| \| --- \| --- \| \| ⬜ \| to assess the effect of *adhering to intervention* (the ‘per-protocol’ effect) \|   **If the aim is to assess the effect of *adhering to intervention***, select the deviations from intended intervention that should be addressed (at least one must be checked):  ⬜ occurrence of non-protocol interventions  ⬜ failures in implementing the intervention that could have affected the outcome  ⬜ non-adherence to their assigned intervention by trial participants  **Which of the following sources were obtained to help inform the risk-of-bias assessment? (tick as many as apply)**  X Journal article(s) with results of the trial  ⬜ Trial protocol  ⬜ Statistical analysis plan (SAP)  ⬜ Non-commercial trial registry record (e.g. ClinicalTrials.gov record)  ⬜ Company-owned trial registry record (e.g. GSK Clinical Study Register record)  ⬜ “Grey literature” (e.g. unpublished thesis)  ⬜ Conference abstract(s) about the trial  ⬜ Regulatory document (e.g. Clinical Study Report, Drug Approval Package)  ⬜ Research ethics application  ⬜ Grant database summary (e.g. NIH RePORTER or Research Councils UK Gateway to Research)  ⬜ Personal communication with trialist  ⬜ Personal communication with the sponsor |
| --- | --- | --- | --- | --- | --- | --- | --- | --- | --- | --- | --- | --- | --- | --- | --- | --- | --- | --- | --- | --- |

## Risk of bias assessment

Responses underlined in green are potential markers for low risk of bias, and responses in red are potential markers for a risk of bias. Where questions relate only to sign posts to other questions, no formatting is used.

**Domain 1: Risk of bias arising from the randomization process**

| **Signalling questions** | **Comments** | **Response options** |
| --- | --- | --- |
| **1.1 Was the allocation sequence random?** |  | Y |
| **1.2 Was the allocation sequence concealed until participants were enrolled and assigned to interventions?** |  | Y |
| **1.3 Did baseline differences between intervention groups suggest a problem with the randomization process?** |  | N |
| **Risk-of-bias judgement** |  | Low |
| Optional: What is the predicted direction of bias arising from the randomization process? |  | NA |

Domain 2: Risk of bias due to deviations from the intended interventions (*effect of assignment to intervention*)

| **Signalling questions** | **Comments** | **Response options** |
| --- | --- | --- |
| **2.1. Were participants aware of their assigned intervention during the trial?** |  | N |
| **2.2. Were carers and people delivering the interventions aware of participants' assigned intervention during the trial?** |  | Y |
| **2.3. If Y/PY/NI to 2.1 or 2.2: Were there deviations from the intended intervention that arose because of the trial context?** |  | N |
| **2.4 If Y/PY to 2.3: Were these deviations likely to have affected the outcome?** |  | NA |
| **2.5. If Y/PY/NI to 2.4: Were these deviations from intended intervention balanced between groups?** |  | NA |
| **2.6 Was an appropriate analysis used to estimate the effect of assignment to intervention?** |  | Y |
| **2.7 If N/PN/NI to 2.6: Was there potential for a substantial impact (on the result) of the failure to analyse participants in the group to which they were randomized?** |  | NA |
| **Risk-of-bias judgement** |  | Some concerns |
| Optional: What is the predicted direction of bias due to deviations from intended interventions? |  | NA |

Domain 2: Risk of bias due to deviations from the intended interventions (*effect of adhering to intervention*)

| **Signalling questions** | **Comments** | **Response options** |
| --- | --- | --- |
| **2.1. Were participants aware of their assigned intervention during the trial?** |  | N |
| **2.2. Were carers and people delivering the interventions aware of participants' assigned intervention during the trial?** |  | Y |
| **2.3. [If applicable:] If Y/PY/NI to 2.1 or 2.2: Were important non-protocol interventions balanced across intervention groups?** |  | Y |
| **2.4. [If applicable:] Were there failures in implementing the intervention that could have affected the outcome?** |  | N |
| **2.5. [If applicable:] Was there non-adherence to the assigned intervention regimen that could have affected participants’ outcomes?** |  | N |
| **2.6. If N/PN/NI to 2.3, or Y/PY/NI to 2.4 or 2.5: Was an appropriate analysis used to estimate the effect of adhering to the intervention?** |  | NA |
| **Risk-of-bias judgement** |  | Some concerns |
| Optional: What is the predicted direction of bias due to deviations from intended interventions? |  | NA |

Domain 3: Missing outcome data

| **Signalling questions** | **Comments** | **Response options** |
| --- | --- | --- |
| **3.1 Were data for this outcome available for all, or nearly all, participants randomized?** |  | Y |
| **3.2 If N/PN/NI to 3.1: Is there evidence that the result was not biased by missing outcome data?** |  | NA |
| **3.3 If N/PN to 3.2: Could missingness in the outcome depend on its true value?** |  | NA |
| **3.4 If Y/PY/NI to 3.3: Is it likely that missingness in the outcome depended on its true value?** |  | NA |
| **Risk-of-bias judgement** |  | Low |
| Optional: What is the predicted direction of bias due to missing outcome data? |  | NA |

Domain 4: Risk of bias in measurement of the outcome

| **Signalling questions** | **Comments** | **Response options** |
| --- | --- | --- |
| **4.1 Was the method of measuring the outcome inappropriate?** |  | N |
| **4.2 Could measurement or ascertainment of the outcome have differed between intervention groups?** |  | N |
| **4.3 If N/PN/NI to 4.1 and 4.2: Were outcome assessors aware of the intervention received by study participants?** |  | Y |
| **4.4 If Y/PY/NI to 4.3: Could assessment of the outcome have been influenced by knowledge of intervention received?** |  | N |
| **4.5 If Y/PY/NI to 4.4: Is it likely that assessment of the outcome was influenced by knowledge of intervention received?** |  | PN |
| **Risk-of-bias judgement** |  | Some Concerns |
| Optional: What is the predicted direction of bias in measurement of the outcome? |  | NA / Favours experimental / Favours comparator / Towards null /Away from null / Unpredictable |

Domain 5: Risk of bias in selection of the reported result

| **Signalling questions** | **Comments** | **Response options** |
| --- | --- | --- |
| **5.1 Were the data that produced this result analysed in accordance with a pre-specified analysis plan that was finalized before unblinded outcome data were available for analysis?** |  | Y |
| **Is the numerical result being assessed likely to have been selected, on the basis of the results, from...** |  |  |
| **5.2. ... multiple eligible outcome measurements (e.g. scales, definitions, time points) within the outcome domain?** |  | N |
| **5.3 ... multiple eligible analyses of the data?** |  | N |
| **Risk-of-bias judgement** |  | Low |
| Optional: What is the predicted direction of bias due to selection of the reported result? |  | NA |

Overall risk of bias

| **Risk-of-bias judgement** |  | High |
| --- | --- | --- |
| Optional: What is the overall predicted direction of bias for this outcome? |  |  |


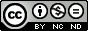


This work is licensed under a [Creative Commons Attribution-NonCommercial-NoDerivatives 4.0 International License](http://creativecommons.org/licenses/by-nc-nd/4.0/).
