# Supplementary material for: Prevalence and characteristics of acute ischemic stroke and intracranial hemorrhage in patients with immune thrombocytopenic purpura and immune thrombotic thrombocytopenic purpura: a systematic review and meta-analysis
Source: Neurol Res Pract. 2025 Mar 17;7(1):19. doi: 10.1186/s42466-025-00374-3 (PMC11921978; doi:10.1186/s42466-025-00374-3)
Supplement: Supplementary file 3 — Supplementary Material 3 [file 42466_2025_374_MOESM3_ESM.docx]

**Supplemental Table 1:** Demographics and Clinical Characteristics of patients with ITP treated with TPO-RAs

**
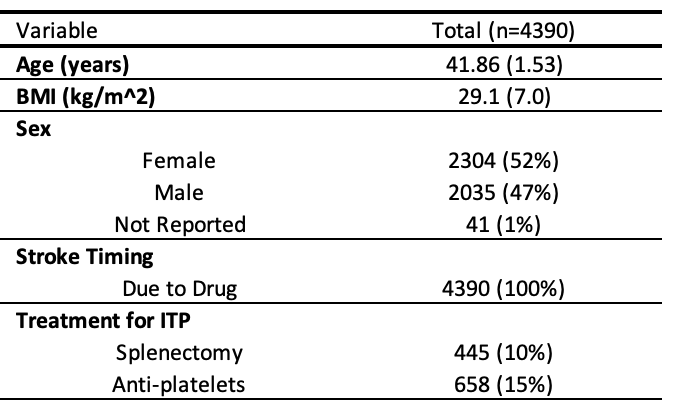

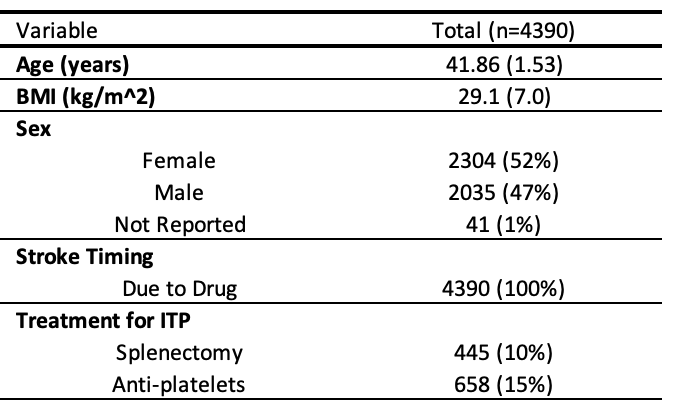
**

BMI: Body mass index. Continuous variables reported as mean (standard deviation). Categorical variables count (percentage).

**Supplemental Table 2:** Meta-regression analysis for risk factors associated with prevalence of stroke based on pre-specified patient’s variables.

**
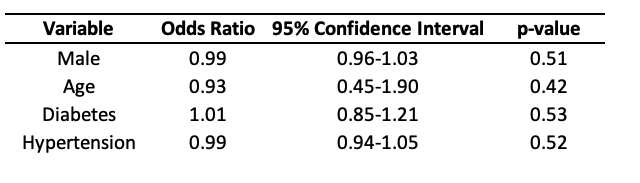
**
