# Supplementary material for: Prevalence and characteristics of acute ischemic stroke and intracranial hemorrhage in patients with immune thrombocytopenic purpura and immune thrombotic thrombocytopenic purpura: a systematic review and meta-analysis
Source: Neurol Res Pract. 2025 Mar 17;7(1):19. doi: 10.1186/s42466-025-00374-3 (PMC11921978; doi:10.1186/s42466-025-00374-3)
Supplement: Supplementary file 4 — Supplementary Material 4 [file 42466_2025_374_MOESM4_ESM.docx]

Dear Dr. Hacke

We are grateful for the ongoing consideration of our paper “Prevalence and Characteristics of Acute Ischemic Stroke and Intracranial Hemorrhage in patients with Immune Thrombocytopenia Purpura and Immune Thrombotic Thrombocytopenic Purpura: A Systematic Review and Meta-Analysis” for publication in Neurological Research and Practice. We are appreciative for the new suggestions to improve our work, and we are providing our point-by-point response to the reviewer comments below:

Reviewer #3

1) "The prevalence of AIS, ICH, and total stroke was lower in all ITP patients when compared to iTTP patients. Additionally, the frequency of stroke did not significantly differ between all ITP patients and ITP patients who experienced stroke as an AE following TPO-RA administration."

- Recommendation: AIS (2.1%) was a more frequent complication than ICH (1.5%) in ITP patients, whereas iTTP patients had a significantly higher prevalence of AIS (13.9%) and ICH (3.9%) compared to ITP patients. Notably, stroke prevalence did not differ significantly between ITP patients treated with TPO-RAs and the overall ITP cohort, suggesting no increased stroke risk associated with TPO-RA use.

Response: Thank you for this suggestion. We have used your recommended language and made the edits in the discussion section. Thank you.

(2) "The mechanism for thrombosis leading to AIS may best be explained by the presence of platelet microparticles (PMPs). These play a role in the formation of atherosclerosis, plaque rupture, and subsequent ischemia. Notably, there are elevated levels of PMPs in patients with ITP who have AIS compared to healthy controls. Furthermore, patients with ITP are known to have prothrombotic phenotypes due to elevated levels of coagulation factors and preactivated platelets. Certain ITP treatments including splenectomy and corticosteroids if used long-term may further increase this risk."

- Recommendation: The development of AIS in ITP patients may be explained by platelet microparticles (PMPs), which contribute to atherosclerosis, plaque rupture, and ischemia. Studies have shown that ITP patients with AIS have significantly higher PMP levels than healthy controls. In addition, ITP is associated with a prothrombotic phenotype due to elevated coagulation factors and preactivated platelets. Certain treatments, such as splenectomy and long-term corticosteroid use, may further amplify this risk.

Response: Thank you for your thoughtful suggestion. We have used your recommended language and made the edits in the discussion section. Indeed, your recommendation is better than what we had.

(3) "AIS (1.8%) and ICH (2.0%) prevalence in ITP patients treated with TPO-RAs was comparable to the overall ITP cohort, suggesting no significant increase in stroke risk due to TPO-RAs. To our knowledge, this is the first study demonstrating a comparable stroke prevalence between these patient populations. These drugs work by activating TPO receptors on megakaryocytes, hematopoietic stem cells, and platelets to stimulate platelet production. Interestingly, the most frequently reported AE is rebound thrombocytopenia—in which a transient increase in platelet count is followed by a drop below normal levels. Although there has been a lot of concern regarding the occurrence of thrombosis for ITP patients treated with TPO-RAs, there has been no clear evidence to suggest an increased risk. Our data supports this notion, and TPO-RAs should continue to be used as a mainstay in ITP treatment from a thromboembolic perspective. However, in clinical practice, extra consideration for

comorbidities should still be given when considering high-risk patients for TPO-RAs."

- This paragraph mixes multiple key findings in one long block, making it quite hard to follow, while some concepts ("Rebound thrombocytopenia") are introduced abruptly. You should try to split this complex, multi-idea sentences.

Response: Thank you for this suggestion. We agree the paragraph mixes multiple key findings making it hard to follow. We removed less relevant concepts such as rebound thrombocytopenia and tried to make the paragraph more focused. The final version reads as such:

- “AIS (1.8%) and ICH (2.0%) prevalence in ITP patients treated with TPO-RAs was comparable to the overall ITP cohort, suggesting no significant increase in stroke risk due to TPO-RAs. These drugs work by activating TPO receptors on megakaryocytes, hematopoietic stem cells, and platelets to stimulate platelet production [(12)](https://www.zotero.org/google-docs/?9Aw3rV). Although there has been concern regarding thrombosis associated with TPO-RAs, there has been no clear evidence to suggest an increased risk [(32)](https://www.zotero.org/google-docs/?voiXvI). Our data supports this notion, and TPO-RAs should continue to be used as a mainstay in ITP treatment from a thromboembolic perspective. However, in clinical practice, extra consideration for comorbidities should still be given when considering high-risk patients.”

(4) The prevalence of ICH in all ITP patients (1.5%) in our study is in accordance with what was reported in a 2015 systematic review on a similar topic [n=1,896 patients, weighted proportion of 1.4% (95% CI 0.9%-2.1%)]. ITP may predispose patients to ICH due to the fact that a low platelet count can lead to disruption of vascular hemostasis, resulting in excess bleeding. Interestingly, our cohort of ITP patients had an average platelet count of 67.60 x 109/L [vs. 47.54 x 109/L in iTTP patients] and experienced a significantly lower prevalence of ICH (1.5% vs. 3.9%, p=0.03) compared to iTTP patients. However, a systematic review (n=10,908) has reported that studies have not shown a consistent association between bleeding and platelet count. More investigation is needed to understand the pathophysiology predisposing ITP patients to ICH.

-> The main finding is burried with unnessery numbers and comparisons, the association between platetel count and ICH risk is unclear - some party are contradictary.

Response: Thank you for providing this point. We have tried to focus the paragraph on the numbers/comparisons that are the most important. Additionally, our goal was to highlight that the association between platelet count and ICH risk is unclear. We were not trying to contradict ourselves, but rather say the data remains inconclusive. We have tried to edit the paragraph to make this point clearer. The final version reads as such:

- “The prevalence of ICH in all ITP patients (1.5%) in our study is in accordance with what was reported in a prior systematic review on a similar topic [n=1,896 patients, weighted proportion of 1.4% [(28)](https://www.zotero.org/google-docs/?cY1PFv). ITP may predispose patients to ICH due to the fact that a low platelet count can lead to disruption of vascular hemostasis [(29)](https://www.zotero.org/google-docs/?7UApvy), resulting in excess bleeding. In accordance with this, our cohort of ITP patients had a greater average platelet count than iTTP patients and experienced a significantly lower prevalence of ICH. However, the data is not completely conclusive, as a prior systematic review reported no consistent association between bleeding and platelet count [(28)](https://www.zotero.org/google-docs/?UMOMtO). For this reason, more investigation is needed to determine the link, if any, between platelet count and ICH risk.”

(5) The use of antithrombotic therapy in iTTP management is controversial. While these agents may carry an increased risk of bleeding, there are also studies demonstrating their efficacy in treatment for patients with iTTP. Only a small percentage of patients with iTTP in our study received antiplatelets (2.3%), and no patients received anticoagulants. While the relatively high prevalence of AIS may support the use of these agents in iTTP management, our results show that iTTP patients are also susceptible to ICH (3.9%). Thus, the use of these agents should be considered with caution."

--> The main point remains unclear -should antithormbotics be used?

- Recommendation: The role of antithrombotic therapy in iTTP remains controversial. While these agents may reduce thrombotic complications, they also pose a bleeding risk, particularly given the 3.9% ICH prevalence observed in our study. Despite the high AIS prevalence (13.9%), only 2.3% of iTTP patients in our dataset received antiplatelet therapy, and none received anticoagulants. This may reflect physician hesitancy due to bleeding concerns. However, emerging evidence suggests that carefully selected patients may benefit from controlled antithrombotic use. Further research is needed to determine whether individualized antithrombotic strategies—accounting for stroke risk factors, platelet counts, and endothelial function—could safely reduce AIS risk without increasing ICH incidence.

Response: Thank you for this suggestion. We have used your recommended language and made the edits in the discussion section. In line with a previous suggestion from another reviewer, we left in how many patients received caplacizumab and plasmapheresis. The final version reads as such:

- “The role of antithrombotic therapy in iTTP remains controversial. While these agents may reduce thrombotic complications [(34–36)](https://www.zotero.org/google-docs/?pp5tHc), they also pose a bleeding risk, particularly given the 3.9% ICH prevalence observed in our study. Despite the high AIS prevalence (13.9%), only 2.3% of iTTP patients in our dataset received antiplatelet therapy, and none received anticoagulants. This may reflect physician hesitancy due to bleeding concerns. However, emerging evidence suggests that carefully selected patients may benefit from controlled antithrombotic use. Similarly, few patients received caplacizumab (2%) or plasmapheresis (13%)—agents that can help to reduce platelet adhesion and remove autoantibodies. Further research is needed to determine whether individualized antithrombotic and antiplatelet strategies—accounting for stroke risk factors, platelet counts, and endothelial function—could safely reduce AIS risk without increasing ICH incidence.”
